# Therapeutic potential of modified Yukgunja-tang (Liujunzi Decoction, Rikkunshito) as an adjuvant treatment for lung cancer: a systematic review and meta-analysis

**DOI:** 10.3389/fphar.2025.1657423

**Published:** 2025-10-01

**Authors:** Sung-Woo Kang, Seojung Ha, Kwan-Il Kim, Hee-Jae Jung, Beom-Joon Lee

**Affiliations:** ^1^ Department of Korean Medicine, College of Korean Medicine, Kyung Hee University, Seoul, Republic of Korea; ^2^ Department of Acupuncture and Moxibustion Medicine, College of Korean Medicine, Sangji University, Wonju, Republic of Korea; ^3^ Division of Allergy, Immune and Respiratory System, Department of Internal Medicine, College of Korean Medicine, Kyung Hee University, Kyung Hee University Medical Center, Seoul, Republic of Korea

**Keywords:** Yukgunja-tang, Liujunzi Decoction, Rikkunshito, herbal medicine, lung cancer, systematic review

## Abstract

**Background:**

Yukgunja-tang (YGJT), also known as Liujunzi Decoction or Rikkunshito, is a traditional East Asian herbal formula widely used to manage symptoms associated with cancer and chemotherapy. This study aimed to systematically evaluate the efficacy and safety of modified YGJT combined with standard antitumor therapy in patients with lung cancer.

**Methods:**

A comprehensive search was conducted in 10 databases through March 2025. Randomized controlled trials comparing modified YGJT plus antitumor therapy versus antitumor therapy alone or placebo were included. Studies involving other herbal combinations or East Asian therapies were excluded. Risk of bias was assessed using the Cochrane Risk of Bias 2 tool. A random-effects model was used for meta-analysis.

**Results:**

Thirty-one trials involving 2,496 participants were included. Modified YGJT significantly improved the objective response rate (ORR; RR 1.69, 95% CI 1.41–2.04), disease control rate (DCR; RR 1.21, 95% CI 1.11–1.31), and Karnofsky Performance Status (KPS; RR 1.79, 95% CI 1.23–2.60; MD 8.62, 95% CI 3.86–13.38). Symptom relief was observed (RR 1.52, 95% CI 1.25–1.85; MD -10.87, 95% CI -12.51 to −9.22), along with improvements in immune markers (CD3^+^, CD4^+^, CD8^+^, CD4+/CD8+ ratio) and reductions in tumor markers (CEA, CYFRA 21-1, NSE, SCC, CA19-9) and adverse events (myelosuppression, leukopenia, gastrointestinal reactions).

**Conclusion:**

Modified YGJT may offer clinical benefits as an adjuvant to standard lung cancer therapy by improving treatment outcomes and reducing toxicity. Large-scale, high-quality trials are needed to confirm these findings.

**Systematic Review Registration:**

https://www.crd.york.ac.uk/PROSPERO/view/CRD42024619038, identifier CRD42024619038.

## Graphical Abstract

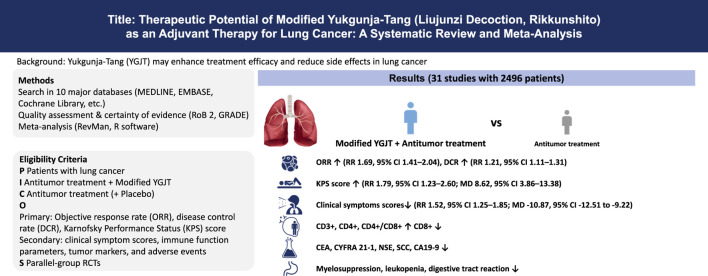

## 1 Introduction

Globally, lung cancer remains the leading cause of cancer-related deaths (Leiter et al., 2023). Although considerable advances have been made in its treatment, including surgery, radiotherapy, chemotherapy, immunotherapy, targeted therapy, and cellular therapies (Lee et al., 2023; Riely et al., 2024), challenges persist in the management of advanced lung cancer (Lee et al., 2023; Riely et al., 2024). Consequently, various complementary and alternative medicine therapies, including traditional herbal medicines, are increasingly being used not only to alleviate cancer-associated symptoms but also to enhance the efficacy of standard treatments and reduce adverse effects, with growing interest and supporting evidence (Guerra-Martin et al., 2021; Li et al., 2021).

Yukgunja-tang (YGJT, also known as Liujunzi Decoction in Chinese and Rikkunshito in Japanese) is a widely used herbal formula in Asia for improving gastrointestinal symptoms, including functional dyspepsia. YGJT was first documented in 1345 during the Yuan dynasty in the classical medical text *Shi Yi De Xiao Fang* (*Efficacious Remedies of Physicians*), authored by Yilin (2009). It comprises six primary medicinal herbs: Ginseng Radix et Rhizoma (*Panax ginseng* C.A.Mey.), Atractylodis macrocephalae Rhizoma (*Atractylodes macrocephala* Koidz.), Poria (*Macrohyporia cocos* (Schwein.) I.Johans. & Ryvarden), Glycyrrhizae Radix et Rhizoma (*Glycyrrhiza uralensis* Fisch. ex DC.), Pinelliae Rhizoma (*Pinellia ternata* (Thunb.) Makino), and Citri Reticulatae Pericarpium (*Citrus reticulata* Blanco) (Inokuchi et al., 2021). In traditional East Asian medicine, YGJT is believed to tonify qi, strengthen the spleen, and eliminate dampness, primarily for treating spleen and stomach qi deficiency accompanied by dampness (Wu et al., 2022). This pattern presents with symptoms such as dyspepsia, fatigue, and weakness, which frequently occur in individuals receiving conventional cancer treatments.

In traditional East Asian medicine, the principle of reinforcing healthy qi to eliminate pathogenic factors constitutes a core therapeutic rationale in the management of cancer (Liu et al., 2018). Lung cancer is defined as a pattern of qi deficiency of the spleen and lung with concomitant phlegm-damp accumulation and secondary impairment of spleen function (You, 2016). Therapeutic strategies focus on strengthening the spleen, supplementing qi, transforming phlegm, and eliminating stasis, and also address chemotherapy-induced impairment of vital qi and gastrointestinal function by reinforcing healthy qi and restoring spleen-stomach integrity (Zhou et al., 2015).

YGJT and its modified formulations are widely prescribed during cancer treatment in East Asian countries (Iwase et al., 2012; Li et al., 2018). A systematic review published in 2023 identified YGJT as the most frequently used herbal intervention for cancer-related anorexia, reflecting its traditional indication for spleen-stomach deficiency and its capacity to address qi deficiency (Park et al., 2023). Moreover, in patients with lung cancer, the adjunctive use of YGJT alongside standard therapies has been associated with improved prognosis, delayed disease progression, and prolonged survival (Liu et al., 2020; Xi et al., 2025). These clinical benefits are consistent with traditional medical theory, which attributes lung cancer to qi deficiency of the spleen and lung and recommends reinforcing qi and resolving phlegm (Li et al., 2017). Through this mechanism, YGJT supports its role as an effective adjunct to standard therapy in lung cancer management.

Although several systematic reviews of randomized controlled trials (RCTs) have examined the efficacy of YGJT and its modified formulations in gastrointestinal disorders, no review has specifically focused on its role in lung cancer (Ko et al., 2021; Sun et al., 2024; Wang et al., 2024). In lung cancer management, YGJT may alleviate cancer- and chemotherapy-related symptoms and improve the effectiveness of anticancer therapies. Therefore, a systematic evaluation of its efficacy and safety is warranted to guide its appropriate integration into clinical practice. This review aims to assess the efficacy and safety of YGJT as an adjunct therapy for individuals with lung cancer and to provide evidence supporting its clinical use.

## 2 Materials and methods

This systematic review was registered with the International Prospective Register of Systematic Reviews (PROSPERO; registration number: CRD42024619038) to ensure transparency and alignment with established review standards. No amendments have been made since registration. The review followed the Preferred Reporting Items for Systematic Reviews and Meta-Analyses (PRISMA) guidelines (Page et al., 2021), the PRISMA extension for Chinese Herbal Medicines 2020 (Zhang et al., 2020), and the Cochrane Handbook for Systematic Reviews of Interventions (Higgins et al., 2024) to maintain methodological rigor. The PRISMA Checklist is provided in Supplementary Table S1.

### 2.1 Search strategy

The final systematic literature search was completed on 3 March 2025, across the following electronic databases: MEDLINE via PubMed, Cochrane Library, EMBASE, Oriental Medicine Advanced Searching Integrated System, Korean Studies Information Service System, Research Information Sharing Service, ScienceON, Korean Medical Database, China National Knowledge Infrastructure Database, and Citation Information by Nii. No language restrictions were applied to minimize selection bias and ensure comprehensive inclusion. The search terms included “lung cancer,” “Yukgunja-Tang,” and “RCT,” along with their synonyms. The comprehensive search strategy, including the exact search strings with all keywords and Boolean operators for each of the 10 databases, is provided in Supplementary Table S2.

### 2.2 Eligibility criteria

#### 2.2.1 Participants

Eligible studies included patients diagnosed with lung cancer. No restrictions were applied regarding baseline characteristics such as age or sex.

#### 2.2.2 Intervention and control

Interventions comprised studies investigating YGJT and its modified formulations. The original formulation included Ginseng Radix et Rhizoma (*P. ginseng* C. A. Mey.), Atractylodis Macrocephalae Rhizoma (*A. macrocephala* Koidz.), Poria (*M. cocos* (Schwein.) I.Johans. & Ryvarden), Glycyrrhizae Radix et Rhizoma (*G. uralensis* Fisch. ex DC.), Pinelliae Rhizoma (*P. ternata* (Thunb.) Makino), and Citri Reticulatae Pericarpium (*C. reticulata* Blanco). Commonly used herbal substitutes in traditional East Asian medicine were also considered; for example, Codonopsis Radix (*Codonopsis pilosula* (Franch.) Nannf.) or Pseudostellariae Radix (*Pseudostellaria heterophylla* (Miq.) Pax) may replace Ginseng Radix et Rhizoma, and Atractylodis Rhizoma (*Atractylodes lancea* (Thunb.) DC.) may substitute Atractylodis Macrocephalae Rhizoma.

Modified YGJT was defined as any prescription that included the six core herbs of YGJT (or their accepted substitutes) plus one or more additional herbs. Variants included modified Hyangsayukgunja-tang (HSYGJT), modified Jigilyukgunja-tang (JGYGJT), and modified Jichulyukgunja-tang (JCYGJT).

The intervention group received modified YGJT, described as “Yukgunja,” “Liujunzi,” or “Rikkunshi,” in combination with antitumor therapies such as surgery, immunotherapy, targeted therapy, chemotherapy, radiotherapy, or cellular therapies, with or without symptom management medications. The control group received either a placebo version of modified YGJT in addition to antitumor therapy or antitumor therapy alone, with symptom management medications, as applicable. Studies using other herbal medicines or traditional East Asian therapies in either group were excluded.

#### 2.2.3 Outcomes

Primary outcomes included objective response rate (ORR), disease control rate (DCR), and Karnofsky Performance Status (KPS) scores. ORR was defined as the proportion of patients achieving complete or partial response, and DCR as the proportion achieving complete response, partial response, or stable disease, based on the Response Evaluation Criteria in Solid Tumors (Wilson et al., 2015). KPS was assessed on a scale from 100 (normal functioning) to 0 (death) in 10-point intervals (Karnofsky, 1949). Changes in KPS were categorized as follows: improvement (≥10-point increase), deterioration (≥10-point decrease), and stable (±10-point change). The improvement rate was calculated as the proportion of patients with improved KPS out of the total. Secondary outcomes included clinical symptom scores; immune function indicators (CD3^+^, CD4^+^, CD8^+^, CD4+/CD8+ ratio, and natural killer cells); tumor markers (including carcinoembryonic antigen (CEA), cytokeratin 19 fragment 21-1 (CYFRA 21-1), neuron-specific enolase (NSE), squamous cell carcinoma antigen (SCC), and carbohydrate antigen 19-9 (CA19-9)); and adverse events such as myelosuppression, leukopenia, and digestive tract reactions. Clinical symptom scores were evaluated as both dichotomous and continuous variables. The dichotomous outcome was measured using the total effective rate, calculated as the percentage of participants categorized as cured, markedly effective, or improved, divided by the total number of participants. The continuous outcome was assessed using the Traditional East Asian Medicine Symptom Score, in accordance with the Guidelines for Clinical Research on New Chinese Medicine published by the Ministry of Health of the People’s Republic of China (Ministry of Health of the People’s Republic of China, 2002). For digestive tract reactions reported as separate symptoms (e.g., nausea and vomiting), the maximum reported prevalence was used as a conservative estimate to avoid overestimation due to symptom overlap (Stewart et al., 2020). A meta-analysis of adverse events was conducted when at least two studies reported data for the same outcome. All outcomes were evaluated at the earliest available post-treatment time point. No restrictions were applied regarding the timing of outcome assessment.

#### 2.2.4 Study design

Only RCTs with a parallel design were included. Studies without full-text availability, case reports, case series, reviews, observational studies, non-RCTs, animal studies, and *in vitro* studies were excluded. This restriction to RCTs was implemented to ensure the highest level of evidence on therapeutic efficacy and to minimize the significant risk of confounding and selection bias inherent in other study designs.

### 2.3 Study selection and data extraction

Search results were imported into EndNote 21 (Clarivate, Philadelphia, PA, USA) for reference management. Duplicate records were initially removed using the built-in deduplication feature. Two independent reviewers (KSW and HSJ) screened the titles and abstracts of retrieved studies against predefined inclusion criteria. Full texts of potentially eligible articles were subsequently assessed to determine final eligibility. Reasons for exclusion after full-text review are provided in Supplementary Table S3. Discrepancies between reviewers were resolved through discussion with a third reviewer (LBJ). The complete selection process was documented using a PRISMA flow diagram.

Data extraction was independently performed by the same two reviewers (KSW and HSJ), who collected information on the following variables: study title, first author, publication year, patient age and sex, sample sizes in the experimental and control groups, lung cancer type, TNM stage, intervention characteristics (herbal medicine components and dosing frequency), treatment duration, and outcome measures. All extracted data were cross-checked for accuracy, and inconsistencies were resolved in consultation with the third reviewer (LBJ).

### 2.4 Quality assessment

The risk of bias for each included RCT was assessed independently by two reviewers (KSW and HSJ) using the Cochrane Risk of Bias 2 tool (Higgins et al., 2024). The assessment covered five domains: the randomization process, deviations from intended interventions, missing outcome data, outcome measurement, and selection of the reported results. Disagreements were resolved through discussion with a third reviewer (LBJ).

### 2.5 Certainty of evidence

The certainty of evidence for each outcome was independently evaluated by two reviewers (KSW and HSJ) using the Grading of Recommendations Assessment, Development, and Evaluation (GRADE) approach. In cases of disagreement, consensus was achieved through discussion with a third reviewer (LBJ). A Summary of Findings table was generated using the GRADEpro Guideline Development Tool (GRADEpro GDT, 2025). The certainty of evidence was categorized as very low, low, moderate, or high, based on considerations including risk of bias, inconsistency, indirectness, imprecision, and publication bias.

### 2.6 Statistical analysis

Meta-analyses were conducted using Review Manager version 5.4 (Cochrane Collaboration), R software version 4.4.0 (R Foundation for Statistical Computing, Vienna, Austria), and the “metafor” package version 4.6.0 (Viechtbauer, 2010). Risk ratios (RRs) were calculated for dichotomous outcomes, and mean differences (MDs) for continuous outcomes, both with 95% confidence intervals (CIs). All included studies provided complete and extractable data; thus, no imputation or transformation of summary statistics was necessary. For outcomes reported in 10 or more studies, potential publication bias was assessed through visual inspection of funnel plots generated using Review Manager 5.4, and formally with Egger’s test, which was conducted using the “metafor” package in R. A random-effects model was applied for all meta-analyses. This choice was made *a priori* due to the anticipated clinical heterogeneity stemming from variations in the modified YGJT, patient populations, and treatment durations across studies. This model was therefore considered more appropriate for providing a conservative estimate of the overall treatment effect. Forest plots were generated using Review Manager 5.4.

### 2.7 Subgroup analysis and assessment of heterogeneity

Pre-specified subgroup analyses were conducted based on the type of anti-tumor therapy, YGJT formulation, comparator group (placebo vs. no additional treatment), cancer stage (early vs. advanced), and lung cancer type (non-small cell lung cancer [NSCLC] vs. small cell lung cancer [SCLC]). Subgroup analyses were performed only when at least two studies were available for a given comparison. Heterogeneity was assessed by visual inspection of forest plots and quantified using the I^2^ statistic (Higgins et al., 2003).

### 2.8 Sensitivity analysis

Sensitivity analyses were conducted to evaluate the robustness of the overall findings. Specifically, we examined outcomes in studies involving patients with advanced-stage lung cancer. An additional sensitivity analysis was performed for studies that applied pattern identification as a diagnostic criterion to assess whether this approach influenced treatment outcomes.

## 3 Results

### 3.1 Retrieval results

**FIGURE 1:**
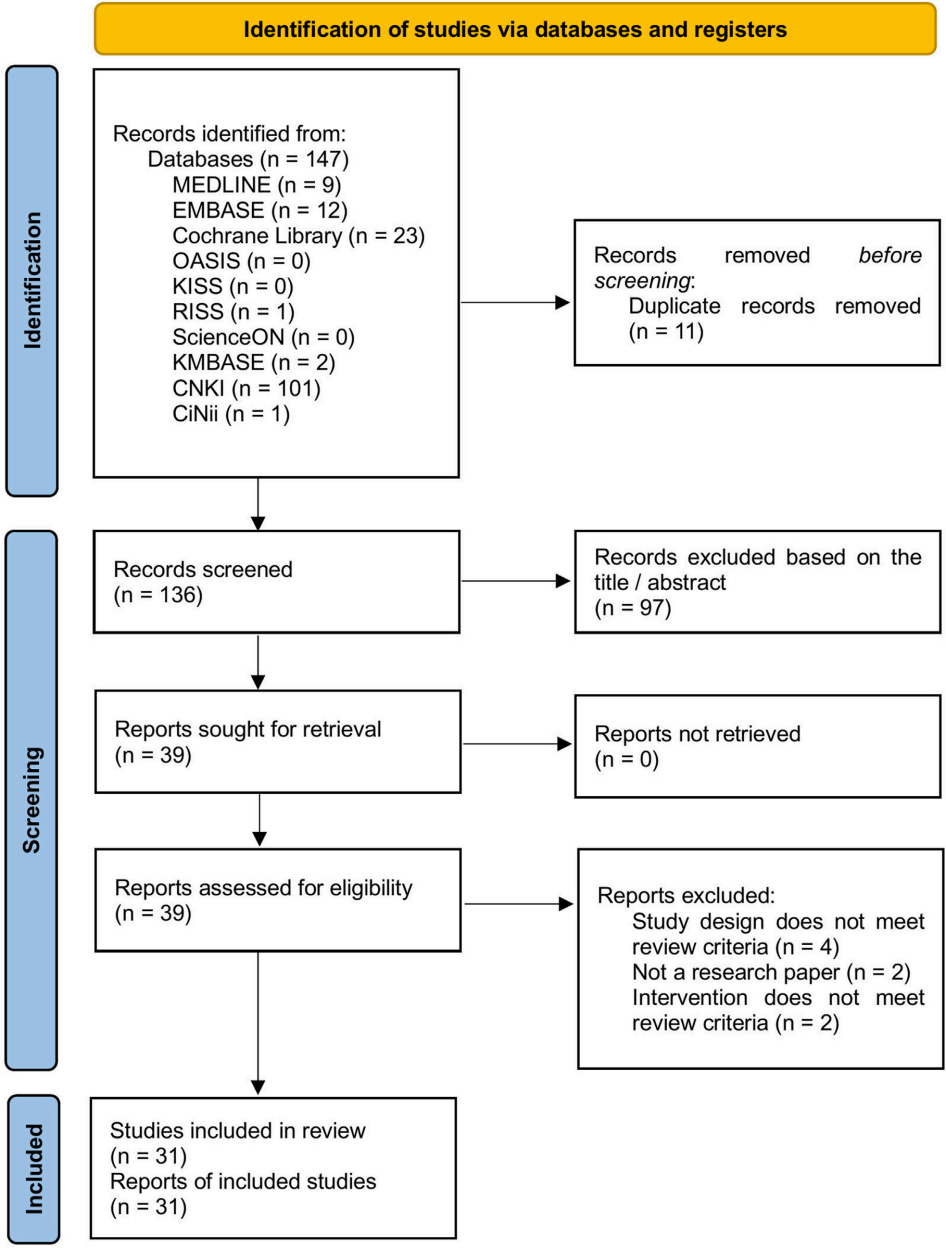
PRISMA flow diagram. CiNii, Citation Information by Nii; CNKI, China National Knowledge Infrastructure Database; KISS, Korean Studies Information Service System; KMBASE, Korean Medical Database; OASIS, Oriental Medicine Advanced Searching Integrated System; RISS, Research Information Sharing Service.

A total of 147 studies were retrieved from the 10 databases. Following the removal of duplicates and screening of titles and abstracts, full-text reviews were conducted for 39 studies to determine eligibility. Eight studies were excluded due to inappropriate study design, non-compliance with the review criteria for the intervention, or lack of original research content. Ultimately, 31 RCTs involving 2,496 participants were included (Figure 1; Supplementary Table S3).

### 3.2 Characteristics of included studies

Table 1 presents the baseline characteristics of the included studies, including first author, publication year, sex, age, sample size, type of lung cancer, TNM stage, intervention details, treatment duration, and outcome measures Table 2; Supplementary Table S4 list the names of the herbal medicines, authors, publication years, principal components, and any modifications.

**TABLE 1:** Baseline characteristics of the 31 randomized controlled trials included in the systematic review.

Author (Year)	Sex (M/F)	Age	Sample size (T/C)	Type of lung cancer	TNM stage	Pattern identification	Treatment group	Control group	Prescription	Duration	Outcome
Fang et al. (2015)	36/24 T: 17/13 C: 19/11	59 ± 18 T: 57 ± 18 (42–75) C: 61 ± 20 (41–74)	60(30/30)	—	—	—	Modified YGJT + DN/DP	DN/DP	1 dose/day, bid, decoction	4 days	WBC count, adverse events (leukopenia, infection), chemotherapy completion rate and delay rate, KPS score
Ye (2015)	33/13 T: 15/8 C: 18/5	47.43 ± 4.36 (34–72) T: 46.79 ± 5.58 C: 48.26 ± 5.24	46(23/23)	—	—	—	Modified YGJT + CT	Omeprazole + CT	1 dose/day, bid, decoction	—	Clinical symptom score
Zhou et al. (2015)	T: 19/12 C: 20/10	T: 58.71 ± 7.23(48–73) C: 60.12 ± 6.81(44–75)	61(31/30)	NSCLC T: ADC (20), SqCC (11) C: ADC (18), SqCC (12)	III-IV T: IIIa (5), IIIb (12), IV (14) C: IIIa (4), IIIb (13), IV (13)	Qi deficiency of the spleen and lung	Modified YGJT + GP/TP/NP	GP/TP/NP	1 dose/day, bid, decoction	12 weeks	ORR (WHO), DCR (WHO), KPS score, clinical symptom score, adverse events (hematological toxicity)
Li et al. (2016)	35/31 T: 18/16 C: 17/15	55.6	66(34/32)	NSCLC - ADC (39), SqCC (27) T: ADC (20), SqCC (14) C: ADC (19), SqCC (13)	IIIb (24), IV (42) T: IIIb (12), IV (22) C: IIIb (12), IV (20)	—	HSYGJT + TP/GP + Ondansetron injection + dexamethasone injection	TP/GP + Ondansetron injection + dexamethasone injection	1 dose/day, qd, decoction	7d	KPS score, adverse events (vomiting)
Xing and Zhai (2016)	T: 20/11 C: 18/13	T: 63.2 ± 7.3 (51–72) C: 58.8 ± 6.4 (46–73)	62(31/31)	Advanced NSCLC T: SqCC (20), ADC (11) C: SqCC (22), ADC (9)	—	—	Modified YGJT + CT (including DP, DN, GP, GN, PP)	CT (including DP, DN, GP, GN, PP)	1 dose/day, bid, decoction	8 weeks	Clinical symptom score, KPS score, CEA
Xiong and Xiong (2016)	T: 20/16 C: 20/16	T: 63 ± 7 (43–72) C: 64 ± 7 (43–72)	72(36/36)	Advanced NSCLC	—	—	Modified YGJT + EP	EP	1 dose/day, bid, decoction	12 weeks	CYFRA 21-1, SCC, NSE, CEA, CA19-9, TUBB3, ERCC-1, MT, p53 protein, Mcll protein, Fbw7 protein, PDCD5, Nrf2, HIF-1α, GLUT1, EGFR-TKI, GSTs, TSGF, VEGF, CD4^+^CD25+Foxp3+Treg/CD4+T levels
Chen et al. (2017)	T: 7/2 C: 6/2	T: 67.5 ± 8.7 C: 56.7 ± 15	17(9/8)	NSCLC T: ADC (7), SqCC (2) C: ADC (6), SqCC (2)	IIIb-IV T: IIIb (3), IV (6) C: IIIb (6), IV (2)	—	YGJT + GP + granisetron + metoclopramide + dexamethasone	Dry powder placebo + GP + metoclopramide + dexamethasone	1 dose/day, qd, powder extract	median 16 weeks T: 8–24 weeks C: 12–20 weeks	ORR, DCR, frequency of adverse events (anorexia, nausea, vomiting, fatigue, dyspnea, anemia, neutropenia, thrombocytopenia), maximum change in leukocyte, hemoglobin levels and platelet counts, OS in 1, 3years, PFS
Fu and Jiang (2017)	T: 27/18 C: 28/17	T: 53.6 ± 5.2 (46–71) C: 54.8 ± 4.9 (47–69)	90(45/45)	Middle and advanced NSCLC T: SqCC (30), ADC (15) C: SqCC (32), ADC (13)	—	—	Modified YGJT + NP + dexamethasone injection + SC	NP + dexamethasone injection + SC	1 dose/day, bid, decoction	12weeks	Weight change, KPS score
Li et al. (2017)	T: 33/26 C: 31/28	T: 63.75 ± 8.24 (44–76) C: 63.28 ± 7.16 (41–75)	118(59/59)	Advanced NSCLC T: ADC (29), SqCC (26), ASC (4) C: ADC (27), SqCC (29), ASC (3)	T: IIIb (25), IV (34) C: IIIb (29), IV (30)	Qi deficiency of the spleen and lung	Modified YGJT + TP + dexamethasone acetate tablets + benzydamine hydrochloride injection + SC	TP + dexamethasone acetate tablets + benzydamine hydrochloride injection + SC	1 dose/day, bid, decoction	12 weeks	ORR, DCR, CD4^+^, CD8^+^, CD4+/CD8+, CEA, SCC, CYFRA 21-1, adverse events (thrombocytopenia, leukopenia, liver dysfunction, digestive tract reaction)
Liu et al. (2017)	T: 21/16 C: 22/15	T: 64.1 ± 5.7 (44–71) C: 63.5 ± 5.9 (44–70)	74(37/37)	Advanced NSCLC	—	—	Modified YGJT + EP	EP	1 dose/day, bid, decoction	12 weeks	CYFRA 21-1, SCC, NSE, CEA, CA19-9, TUBB3, ERCC-1, MT, P53, Mcll, Fbw7, PDCD5, Nrf2, HIF-1α, GLUT1, CD4^+^CD25+Foxp3+Treg/CD4+T, VEGF, GSTs, TSGF
Yang et al. (2019)	T: 30/17 C: 27/16	T: 56.61 ± 9.82 (32–72) C: 55.81 ± 8.22 (33–73)	90(47/43)	Lung cancer T: ADC (18), SqCC (16), SCLC (11), ASC (2) C: ADC (16), SqCC (15), SCLC (10), ASC (2)	—	—	Modified YGJT + DP (ADC, SqCC, ASC)/EP (SCLC) + SC	DP (ADC, SqCC, ASC)/EP (SCLC) + SC	1 dose/day, -, decoction	3 weeks	CD3, CD4, CD8, CD4/CD8, CD56, IgG, IgM, IgA, lymphocyte percentage, absolute lymphocyte count, adverse events (digestive tract reaction, myelosuppression)
Zhao et al. (2021)	T: 27/14 C: 24/13	T: 52.3 ± 6.5 C: 54.8 ± 7.3	78(41/37)	NSCLC T: ADC (33), SqCC (8) C: ADC (30), SqCC (7)	IV	—	Modified YGJT + CT (including cisplatin or carboplatin + pemetrexed, gemcitabine or docetaxel)	CT (including cisplatin or carboplatin + pemetrexed, gemcitabine or docetaxel)	1 dose/day, bid, decoction	6 weeks	ORR (WHO), DCR (WHO), KPS score, clinical symptom score, adverse events (digestive tract reaction, myelosuppression)
Dong et al. (2018)	T: 23/22 C: 24/21	T: 63.76 ± 2.91 (46–79) C: 63.91 ± 2.24 (45–79)	90(45/45)	Lung cancer	—	—	Modified YGJT + EP	EP	1 dose/day, bid, decoration	6 weeks	Clinical symptom score, median PFS, median OS, GLUT1, PDCD5, adverse events (myelosuppression, digestive tract reaction)
Duan (2018)	T: 23/21 C: 24/20	T: 63.17 ± 5.41 (46–71) C: 63.45 ± 5.26 (45–72)	88(44/44)	Middle and advanced NSCLC T: ADC (26), SqCC (18) C: ADC (27), SqCC (17)	—	—	Modified YGJT + PP + vit B injection + oral folic acid + oral dexamethasone	PP + vit B injection + oral folic acid + oral dexamethasone	1 dose/day, bid, decoction	12 weeks	KPS score, CYFRA 21-1, CEA, NSE, adverse events (headache, leukopenia, nausea and vomiting, myelosuppression)
Harada et al. (2018)	44/13 T: 23/6 C: 21/7	Median 65 (46–76) T: median 66 (55–76) C: median 65 (46–73)	58(29/29)	Lung cancer ADC (31), SCLC (14), SqCC (8) T: ADC (13), SqCC (5), SCLC (9), LCNEC (1) C: ADC (18), SqCC (3), LCC (2), SCLC (5)	—	—	YGJT + CT (cisplatin-based) + SC	CT (cisplatin-based) + SC	7.5 g/day, tid, granule	7d	Complete response rate (emesis, nausea, rescue medication), eating impairment, nausea (VAS), dietary intake (VAS), adverse events (constipation, diarrhea, hiccups)
Harada et al. (2018)	48/14 T: 26/6 C: 22/8	Median 70 (45–89) T: median 71 (49–89) C: median 68 (45–79)	62(32/30)	Lung cancer - ADC (29), SqCC (19), SCLC (8) T: ADC (16), SqCC (10), SCLC (3), LCNEC (2), PC (1) C: ADC (13), SqCC (9), SCLC (5), LCC (1), NS (2)	—	—	YGJT + CT (carboplatin-based) + SC	CT (carboplatin-based) + SC	7.5 g/day, tid, granule	7d	Complete response rate (emesis, nausea, rescue medication), eating impairment, nausea (VAS), dietary intake (VAS), adverse events (constipation, diarrhea, hiccups)
Li (2018)	T: 19/19 C: 20/18	T: 73.12 ± 2.06 (61–78) C: 73.17 ± 2.03 (62–78)	76(38/38)	Lung cancer T: SqCC (21), ADC (17) C: SqCC (20), ADC (18)	—	Qi deficiency with phlegm dampness	Modified YGJT + GP	GP	1 dose/day, bid, decoction	12 weeks	ORR (WHO), DCR (WHO), clinical symptom score
Liu et al. (2018)	T: 26/14 C: 25/15	T: 63.4 ± 2.4 C: 62.8 ± 2.6	80(40/40)	NSCLC	—	Qi deficiency of the spleen and lung	Modified YGJT + GP + SC	GP + SC	1 dose/day, bid, decoction	9 weeks	CD3^+^, CD4^+^, CD4+/CD8+, CD8^+^, NK cell, KPS score, clinical symptom score
Sun (2020)	T: 25/13 C: 24/14	T: 57.57 ± 4.18 (33–75) C: 57.68 ± 4.06 (34–75)	76(38/38)	Advanced NSCLC	—	Qi deficiency of the spleen and lung	Modified YGJT + gemcitabine/paclitaxel/pemetrexed/cisplatin	gemcitabine/paclitaxel/pemetrexed/cisplatin	1 dose/day, bid, decoction	12 weeks	CEA, SCC, CD4, CD8, CD4/CD8
Sun et al. (2021)	T: 17/8 C: 16/9	T: 62.5 ± 2.4 (3.6 ± 1.5) C: 62.9 ± 2.5 (3.5 ± 1.8)	50(25/25)	NSCLC	III-IV	Qi deficiency of the spleen and lung	Modified YGJT + gemcitabine/paclitaxel/pemetrexed/cisplatin	gemcitabine/paclitaxel/pemetrexed/cisplatin	1 dose/day, bid, decoction	12 weeks	VEGF, CD3^+^, CD4^+^, CD8^+^, CD4+/CD8+ NK cell, KPS score, clinical symptom score, ORR, DCR
Zhao et al. (2021)	T: 22/36 C: 21/36	T: 65.59 ± 9.89 C: 63.16 ± 11.85	120(60/60)	NSCLC	IIIb-IV	Spleen deficiency with phlegm dampness	Modified YGJT + gefitinib + SC	gefitinib + SC	1 dose/day, qd, decoction	8–10 m	KPS score, ORR, DCR, median PFS, adverse events (digestive tract reaction)
Cai et al. (2022)	T: 28/17 C: 26/19	T:≥60 (26) C:≥60 (24)	90(45/45)	Lung cancer	T: IIIb (12), IV (33) C: IIIb (14), IV (31)	Qi deficiency of the spleen and lung	JCYGJT + CT + SC	CT + SC	1 dose/day, tid, decoction	6 weeks	The tongue coating thickness scores, FACT-L, adverse events (decreased appetite, nausea, vomiting, diarrhea, constipation)
Chen et al. (2022)	T: 29/20 C: 28/21	T: 57.43 ± 5.39 (44–74) C: 58.14 ± 5.63 (45–73)	98(49/49)	Advanced NSCLC	III-IV	Qi deficiency of the spleen and lung	Modified YGJT + GP	GP	1 dose/day, bid, decoction	6 weeks	CYFRA21-1, CA125, NSE, VEGF, MMP-9, bFGF, PGE2, CD4+/CD8+, CD4^+^, adverse events (digestive tract reaction, myelosuppression, liver and kidney dysfunction, peripheral nerve injury), ORR (WHO), DCR (WHO)
Guo et al. (2024)	T: 24/16 C: 22/18	T: 59.51 ± 7.19 (38–75) C: 59.67 ± 7.21 (39–77)	80(40/40)	NSCLC T: SqCC (18), ADC (15), ASC (7) C: SqCC (19), ADC (16), ASC (5)	T: III (27), IV (13) C: III (28), IV (12)	—	Modified YGJT + GP	GP	1 dose/day, bid, decoction	9 weeks	ORR, DCR, CD3^+^, CD4^+^, CD4+/CD8+, NK cells, KPS score, clinical symptom score
Guo et al. (2024)	T: 25/15 C: 26/14	T: 58.14 ± 16.95 (42–74) C: 57.95 ± 18.24 (39–76)	80(40/40)	Lung cancer T: SCLC (22), SqCC (14), ADC (4) C: SCLC (25), SqCC (9), ADC (6)	—	—	Modified HSYGJT + CT (cisplatin-based) + SC	CT (cisplatin-based) + SC	1 dose/day, bid, decoction	5d	QOL questionnaire, laboratory tests, adverse events (digestive tract reaction, myelosuppression, liver function and kidney function damage)
Nie et al. (2022)	T: 29/11 C: 26/14	T: 67.52 ± 3.52 (60–76) C: 68.41 ± 3.34 (60–76)	80(40/40)	Advanced lung cancer T: SqCC (19), ADC (12), NEC (9) C: SqCC (20), ADC (13), NEC (7)	T: III (26), IV (14) C: III (24), IV (16)	Qi deficiency of the spleen and lung	Modified YGJT + CT (platinum-containing two-drug regimen)	CT (platinum-containing two-drug regimen)	1 dose/day, bid, decoction	6 weeks	CD3^+^, CD4^+^, CD8^+^, CD4+/CD8+, IgM, IgG, IgA, CYFRA 21-1, CA125, NSE, CEA, FACT-L ORR, DCR, adverse events (nausea, vomiting, abdominal pain, diarrhea)
Wang (2022)	T: 24/16 C: 22/18	T: 68.15 ± 3.21 (53–85) C: 68.12 ± 3.15 (55–86)	80(40/40)	Advanced lung cancer T: ADC (28), SqCC (12) C: ADC (26), SqCC (14)	—	—	Modified JGYGJT + TP	TP	1 dose/day, qd, decoction	6 weeks	ORR, DCR, BFI, KPS score, SF-36, CD3^+^, CD4^+^, CD8^+^, CD4+/CD8+, CA19-9, CEA, CA125
Liu et al. (2023)	T: 40/16 C: 36/18	T: 58.17 ± 10.04 C: 59.10 ± 11.05	110(56/54)	SCLC	T: LS (13), ES - single metastasis (14), ES - mixed metastasis (29) C: LS (15), ES - single metastasis (12), ES - mixed metastasis (27)	Qi deficiency of the spleen and stomach	Modified HSYGJT + EP or (irinotecan + cisplatin) + SC (+radiotherapy)	EP or (irinotecan + cisplatin) + SC (+radiotherapy)	—	18 weeks	CFS, clinical symptom score, adverse events (including hyponatremia, digestive tract reaction, hematological toxicity), PFS in 1y
Tao and Liu (2023)	T: 12/18 C: 11/19	T: 60.96 ± 2.94 (51–70) C: 61.36 ± 3.03 (50–69)	60(30/30)	NSCLC T: SqCC (21), ADC (7), ASC (2) C: SqCC (20), ADC (8), ASC (2)	T: II (10), III (20) C: II (9), III (21)	Spleen Qi deficiency	Modified YGJT + leucogen tablet + CT	leucogen tablet + CT	1 dose/day, bid, decoction	2 weeks	Clinical symptom score, WBC count, neutrophil count, CEA, KPS score
Wang et al. (2024)	T: 35/15 C: 34/16	T: 63.2 ± 5.7 (53–73) C: 63.9 ± 6.2 (52–71)	100(50/50)	Advanced NSCLC	—	—	Modified YGJT + GP	GP	1 dose/day, bid, decoction	4 weeks	CYFRA21-1, CEA, IgG, IgA, residual symptoms, median OS in 2y
Bi et al. (2024)	T: 28/27 C: 29/26	T: 58.58 ± 6.10 (43–64) C: 57.80 ± 6.00 (45–65)	110(55/55)	NSCLC	T: IIIb (31), IV (24) C: IIIb (30), IV (25)	Qi deficiency	JCYGJT + GP	GP	1 dose/day, bid, decoction	6 weeks	Clinical symptom score, the tongue coating thickness scores, CFS, CD3^+^, CD4^+^, CD8^+^, CD4+/CD8+, CYFRA21-1, NSE, CA125, CEA, adverse events (digestive tract reaction, liver and kidney dysfunction, myelosuppression)
Lin et al. (2024)	T: 22/15 C: 21/16	T: 60.1 ± 1.9 (46–78) C: 59.5 ± 1.8 (45–77)	74(37/37)	Advanced NSCLC T: ADC (13), SqCC (12), ASC (12) C: SqCC (13), ADC (12), ASC (12)	—	Spleen deficiency with phlegm dampness	Modified YGJT + osimertinib	osimertinib	1 dose/day, bid, decoction	3 m	ORR, DCR, NK cells, CD4^+^, CD8^+^, CD3^+^, HIF-1α, VEGF, QOL

ADC, adenocarcinoma; ASC, adenosquamous carcinoma; bFGF, basic fibroblast growth factor; BFI, brief fatigue inventory; C, control; CA125, cancer antigen 125; CA19-9, carbohydrate antigen 19-9; CEA, carcinoembryonic antigen; CFS, cancer fatigue scale; CT, chemotherapy; CYFRA, 21-1, cytokeratin 19 fragment 21-1; DCR, disease control rate; DN, nedaplatin plus docetaxel; DP, docetaxel plus cisplatin; EGFR-TKI, epidermal growth factor receptor tyrosine kinase inhibitor; EP, etoposide plus cisplatin; ERCC-1, excision repair cross-complementation group 1; FACT-L, Functional Assessment of Cancer Therapy-Lung; Fbw7, F-box and WD, repeat domain-containing 7; Foxp, Forkhead box P; GLUT1, glucose transporter 1; GN, gemcitabine plus nedaplatin; GP, gemcitabine plus cisplatin; GSTs, glutathione S-transferases; HIF-1α, hypoxia-inducible factor 1-alpha; HSYGJT, Hyangsayukgunja-Tang; Ig, immunoglobulin; JCYGJT, Jichulyukgunja-Tang; JGYGJT, Jigilyukgunja-Tang; KPS, karnofsky performance status; LCC, large cell carcinoma; LCNEC, large cell neuroendocrine carcinoma; Mcll, myeloid cell leukemia-1; MMP-9, matrix metalloproteinase-9; MT, metallothionein; NEC, neuroendocrine carcinoma; NK, natural killer; NP, vinorelbine plus cisplatin; Nrf2, nuclear factor erythroid 2–related factor 2; NS, non-specified; NSCLC, non-small cell lung cancer; NSE, neuron-specific enolase; ORR, objective response rate; OS, overall survival; PC, pleomorphic carcinoma; PDCD5, programmed cell death 5; PFS, progression-free survival; PGE2, prostaglandin E2; PP, pemetrexed plus cisplatin; QOL, quality of life; SC, standard care; SCC, squamous cell carcinoma; SCLC, small cell lung cancer; SF-36, 36-Item Short Form Survey; SqCC, squamous cell carcinoma; T, treatment; TP, paclitaxel plus cisplatin; TUBB3, class III, beta-tubulin; TSGF, tumor-specific growth factor; VAS, visual analogue scale; VEGF, vascular endothelial growth factor; WBC, white blood cell; YGJT, Yukgunja-Tang.

**TABLE 2:** Composition of modified YGJT used in the included studies.

Name	Author (Year)	Main components	Modified part
Modified YGJT	Fang et al. (2015)	Codonopsis Radix (*Codonopsis pilosula* (Franch.) Nannf.) 30 g, Atractylodis Macrocephalae Rhizoma (*Atractylodes macrocephala* Koidz.) 10 g, Poria (*Macrohypori*a cocos (Schwein.) I.Johans. & Ryvarden) 15 g, Glycyrrhizae Radix et Rhizoma (*Glycyrrhiza uralensis* Fisch. ex DC.) 3 g, Pinelliae Rhizoma (*Pinellia ternata* (Thunb.) Makino) 9 g, Citri Reticulatae Pericarpium (*Citrus reticulata* Blanco) 10 g, Jujubae Fructus (*Ziziphus jujuba* Mill.) 30 g	Spatholobi Caulis (*Spatholobus suberectus* Dunn) 30 g, Curculiginis Rhizoma (*Curculigo orchioides* Gaertn.) 15 g, Epimedii Folium (*Epimedium brevicornu* Maxim.) 15 g, Ligustri Lucidi Fructus (*Ligustrum lucidum* W.T.Aiton) 30 g, Astragali Radix (*Astragalus mongholicus* Bunge) 30 g, Angelicae Sinensis Radix [*Angelica sinensis* (Oliv.) Diels] 15 g
Modified YGJT	Ye (2015)	Ginseng Radix et Rhizoma (*Panax ginseng* C.A.Mey.) 9 g, Atractylodis Macrocephalae Rhizoma (*Atractylodes macrocephala* Koidz.) 12 g, Poria (*Macrohyporia cocos* (Schwein.) I.Johans. & Ryvarden) 12 g, Glycyrrhizae Radix et Rhizoma (*Glycyrrhiza uralensis* Fisch. ex DC.) 6 g, Citri Reticulatae Pericarpium (*Citrus reticulata* Blanco) 6 g, Pinelliae Rhizoma (*Pinellia ternata* (Thunb.) Makino) 6 g, Zingiberis Rhizoma Recens (*Zingiber officinale* Roscoe) 3 slices	Dioscoreae Rhizoma (*Dioscorea polystachya* Turcz.) 15 g, Bambusae Caulis in Taenias (*Bambusa tuldoides* Munro) 12 g, Massa Medicata Fermentata 12 g, Crataegi Fructus (*Crataegus pinnatifida* Bunge) 15 g 1) Diarrhea: add Adenophorae Radix (*Adenophora triphylla* (Thunb.) A.DC.) 6 g, Portulacae Herba (*Portulaca oleracea* L.) 15 g 2) Constipation: add Trichosanthis Fructus (*Trichosanthes kirilowii* Maxim.) 15 g, Cannabis Fructus (*Cannabis sativa* L.) 6 g 3) Oral inflammation: add Rehmanniae Radix (*Rehmannia glutinosa* (Gaertn.) Libosch. ex DC.) 12 g, Coptidis Rhizoma (*Coptis chinensis* Franch.) 3 g
Modified YGJT	Zhou et al. (2015)	Codonopsis Radix (*Codonopsis pilosula* (Franch.) Nannf.) 30 g, Poria (*Macrohyporia cocos* (Schwein.) I.Johans. & Ryvarden) 15 g, Atractylodis Macrocephalae Rhizoma (*Atractylodes macrocephala* Koidz.) 15 g, Pinelliae Rhizoma (*Pinellia ternata* (Thunb.) Makino) 10 g, Citri Reticulatae Pericarpium (*Citrus reticulata* Blanco)10 g, Glycyrrhizae Radix et Rhizoma (*Glycyrrhiza uralensis* Fisch. ex DC.) 5 g, Jujubae Fructus (*Ziziphus jujuba* Mill.) 10 g, Zingiberis Rhizoma Recens (*Zingiber officinale* Roscoe) 3 slices	During chemotherapy 1) Cough with sputum: add Fritillariae Thunbergii Bulbus (*Fritillaria thunbergii* Miq.) 15 g, Armeniacae Semen Amarum (*Prunus armeniaca* L.) 10 g, Stemonae Radix [*Stemona sessilifolia* (Miq.) Miq.]10 g 2) Constipation: add Rhei Radix et Rhizoma (*Rheum palmatum* L.) 10 g, Aurantii Fructus (*Citrus × aurantium* L.) 10 g 3) Nausea and vomiting: add Inulae Flos (*Inula japonica* Thunb.) 10 g, Haematitum 30 g 4) Significant fatigue: add Ginseng Radix et Rhizoma (*Panax ginseng* C.A.Mey.) 10 g, Astragali Radix (*Astragalus mongholicus* Bunge) 30 g 5) Poor appetite: add Oryzae Fructus Germinatus (*Oryza sativa* L.) 30 g, Hordei Fructus Germinatus (*Hordeum vulgare* L.) 30 g, Galli Gigerii Endothelium Corneum [*Gallus gallus domesticus* (Linnaeus, 1758)] 10 g 6) Leukopenia: add Hominis Placenta 10 g, Ligustri Lucidi Fructus (*Ligustrum lucidum* W.T.Aiton) 15 g 7) Loose stools: add Agastachis Herba [*Agastache rugosa* (Fisch. & C.A.Mey.) Kuntze] 10 g, Amomi Fructus [Wurfbainia villosa (Lour.) Škorničk. & A.D.Poulsen] 3 g During the chemotherapy interval 1) Appropriately select medicines for transforming phlegm, resolving masses, and eliminating stasis: add Solani Nigri Herba (*Solanum nigrum* L.) 20 g, Scolopendra (*Scolopendra subspinipes mutilans* L.Koch, 1878) 4 pieces, Fritillariae Thunbergii Bulbus (*Fritillaria thunbergii* Miq.) 10 g, Prunellae Spica (*Prunella vulgaris* L.) 10 g, Curcumae Rhizoma [*Curcuma phaeocaulis* Valeton) 10 g, Pelodiscis Carapax (*Pelodiscus sinensis* (Wiegmann, 1835)] 10 g, Ranunculi Ternati Radix (*Ranunculus ternatus* Thunb.) 15 g
HSYGJT	Li et al. (2016)	Codonopsis Radix [*Codonopsis pilosula* (Franch.) Nannf.] 15 g, Atractylodis Macrocephalae Rhizoma (*Atractylodes macrocephala* Koidz.) 15 g, Poria (*Macrohyporia cocos* (Schwein.) I.Johans. & Ryvarden) 15 g, Pinelliae Rhizoma [*Pinellia ternata* (Thunb.) Makino] 10 g, Citri Reticulatae Pericarpium (*Citrus reticulata* Blanco) 10 g, Aucklandiae Radix [*Dolomiaea costus* (Falc.) Kasana & A.K.Pandey] 10 g, Amomi Fructus [*Wurfbainia villosa* (Lour.) Škorničk. & A.D.Poulsen] 10 g, Glycyrrhizae Radix et Rhizoma (*Glycyrrhiza uralensis* Fisch. ex DC.) 5 g	-
Modified YGJT	Xing and Zhai (2016)	Codonopsis Radix [*Codonopsis pilosula* (Franch.) Nannf.] 15 g, Atractylodis Macrocephalae Rhizoma (*Atractylodes macrocephala* Koidz.) 10 g, Poria [*Macrohyporia cocos* (Schwein.) I.Johans. & Ryvarden] 10 g, Pinelliae Rhizoma (*Pinellia ternata* (Thunb.) Makino) 10 g, Citri Reticulatae Pericarpium (*Citrus reticulata* Blanco) 10 g, Glycyrrhizae Radix et Rhizoma (*Glycyrrhiza uralensis* Fisch. ex DC.) 3 g	Astragali Radix (*Astragalus mongholicus* Bunge) 15 g, Platycodonis Radix (*Platycodon grandiflorus* (Jacq.) A.DC.) 10 g, Coicis Semen (*Coix lacryma-jobi* var. *ma-yuen* (Rom.Caill.) Stapf) 15 g, Fritillariae Cirrhosae Bulbus (*Fritillaria cirrhosa* D.Don) 5 g, Armeniacae Semen Amarum (*Prunus armeniaca* L.) 10 g
Modified YGJT	Xiong and Xiong (2016)	Codonopsis Radix (*Codonopsis pilosula* (Franch.) Nannf.) 30 g, Poria (*Macrohyporia cocos* (Schwein.) I.Johans. & Ryvarden) 15 g, Atractylodis Macrocephalae Rhizoma (Atractylodes macrocephala Koidz.) 15 g, Pinelliae Rhizoma (*Pinellia ternata* (Thunb.) Makino) 10 g, Citri Reticulatae Pericarpium (*Citrus reticulata* Blanco) 10 g, Glycyrrhizae Radix et Rhizoma (*Glycyrrhiza uralensis* Fisch. ex DC.) 5 g	Inulae Flos (*Inula japonica* Thunb.) 30 g, Fritillariae Thunbergii Bulbus (*Fritillaria thunbergii* Miq.) 15 g, Stemonae Radix (*Stemona sessilifolia* (Miq.) Miq.) 10 g, Aurantii Fructus (*Citrus × aurantium* L.) 10 g, Hominis Placenta 10 g, Armeniacae Semen Amarum (*Prunus armeniaca* L.) 10 g
YGJT	Chen et al. (2017)	Ginseng Radix et Rhizoma (*Panax ginseng* C.A.Mey.) 5 g, Atractylodis Macrocephalae Rhizoma (*Atractylodes macrocephala* Koidz.) 5 g, Poria (*Macrohyporia cocos* (Schwein.) I.Johans. & Ryvarden) 5 g, Pinelliae Rhizoma (*Pinellia ternata* (Thunb.) Makino) 5 g, Glycyrrhizae Radix et Rhizoma (*Glycyrrhiza uralensis* Fisch. ex DC.) 2.5 g, Citri Reticulatae Pericarpium (*Citrus reticulata* Blanco) 2.5 g, Zingiberis Rhizoma Recens (*Zingiber officinale* Roscoe) 2.5 g, Jujubae Fructus (*Ziziphus jujuba* Mill.) 2.5 g, starch 7.8 g per 100.00 g powder with a crude drug extract ratio of 30.00:7.80	-
Modified YGJT	Fu and Jiang (2017)	Codonopsis Radix (*Codonopsis pilosula* (Franch.) Nannf.) 15 g, Atractylodis Macrocephalae Rhizoma (*Atractylodes macrocephala* Koidz.) 15 g, Poria (*Macrohyporia cocos* (Schwein.) I.Johans. & Ryvarden) 15 g, Glycyrrhizae Radix et Rhizoma (*Glycyrrhiza uralensis* Fisch. ex DC.) 10 g, Citri Reticulatae Pericarpium (*Citrus reticulata* Blanco) 20 g, Pinelliae Rhizoma (*Pinellia ternata* (Thunb.) Makino) 12 g	1) Significant pain: add Corydalis Rhizoma (*Corydalis yanhusuo* (Y.H.Chou & Chun C.Hsu) W.T.Wang ex Z.Y.Su & C.Y.Wu), Aristolochiae Herba (*Aristolochia debilis* Siebold & Zucc.) 2) Dysphagia: add Inulae Flos *(Inula japonica* Thunb.), Haematitum 3) Vomiting of acid, phlegm, and saliva: add Coptidis Rhizoma (*Coptis chinensis* Franch.), Euodiae Fructus (*Tetradium ruticarpum* (A.Juss.) T.G.Hartley) 4) Insomnia: add Ziziphi Spinosae Semen (*Ziziphus jujuba* Mill.), Polygoni Multiflori Caulis (*Reynoutria multiflora* (Thunb.) Moldenke)
Modified YGJT	Li et al. (2017)	Codonopsis Radix (*Codonopsis pilosula* (Franch.) Nannf.), Poria (*Macrohyporia cocos* (Schwein.) I.Johans. & Ryvarden) 15 g, Atractylodis Macrocephalae Rhizoma (*Atractylodes macrocephala* Koidz.) 10 g, Pinelliae Rhizoma (*Pinellia ternata* (Thunb.) Makino) 10 g, Tangerine Pith (*Citrus reticulata* Blanco) 10 g, Glycyrrhizae Radix et Rhizoma (Glycyrrhiza uralensis Fisch. ex DC.) 6 g	Astragali Radix (*Astragalus mongholicus* Bunge) 15 g, Polygonati Rhizoma (*Polygonatum kingianum* Collett & Hemsl.) 15 g, Schisandrae Chinensis Fructus (*Schisandra chinensis* (Turcz.) Baill.) 10 g, Platycodonis Radix (*Platycodon grandiflorus* (Jacq.) A.DC.) 10 g, Scutellariae Barbatae Herba (*Scutellaria barbata* D.Don) 30 g, Hedyotidis Herba (*Oldenlandia diffusa* (Willd.) Roxb.) 30 g 1) Severe shortness of breath and coughing due to qi deficiency: add Panacis Quinquefolii Radix (*Panax quinquefolius* L.) 10 g 2) Phlegm, toxin, and stasis accumulation: add Vespae Nidus [*Polistes olivaceus* (de Geer, 1773)] 10 g, Bombyx Batryticatus (*Bombyx mori* (Linnaeus, 1758)) 10 g, Persicae Semen (*Prunus persica* (L.) Batsch) 10 g 3) Nausea and vomiting: add Inulae Flos *(Inula japonica* Thunb.) 15 g, Haematitum 15 g 4) Leukopenia: add Hominis Placenta 10 g, Ligustri Lucidi Fructus (*Ligustrum lucidum* W.T.Aiton) 10 g
Modified YGJT	Liu et al. (2017)	Codonopsis Radix (*Codonopsis pilosula* (Franch.) Nannf.) 30 g, Poria (*Macrohyporia cocos* (Schwein.) I.Johans. & Ryvarden) 15 g, Atractylodis Macrocephalae Rhizoma (*Atractylodes macrocephala* Koidz.) 15 g, Pinelliae Rhizoma (*Pinellia ternata* (Thunb.) Makino) 10 g, Citri Reticulatae Pericarpium (*Citrus reticulata* Blanco) 10 g, Glycyrrhizae Radix et Rhizoma (*Glycyrrhiza uralensis* Fisch. ex DC.) 5 g	Inulae Flos (*Inula japonica* Thunb.) 10 g, Fritillariae Thunbergii Bulbus (*Fritillaria thunbergii* Miq.) 15 g, Stemonae Radix (*Stemona sessilifolia* (Miq.) Miq.) 10 g, Aurantii Fructus (*Citrus × aurantium* L.) 10 g, Hominis Placenta 10 g, Armeniacae Semen Amarum (*Prunus armeniaca* L.) 10 g
Modified YGJT	Yang et al. (2019)	Ginseng Radix et Rhizoma (*Panax ginseng* C.A.Mey.) 15 g, Poria (*Macrohyporia cocos* (Schwein.) I.Johans. & Ryvarden) 30 g, Atractylodis Macrocephalae Rhizoma (*Atractylodes macrocephala* Koidz.) 20 g, Citri Reticulatae Pericarpium (*Citrus reticulata* Blanco) 12 g, Pinelliae Rhizoma (*Pinellia ternata* (Thunb.) Makino) 12 g, Glycyrrhizae Radix et Rhizoma (*Glycyrrhiza uralensis* Fisch. ex DC.) 6 g	Astragali Radix (*Astragalus mongholicus* Bunge) 40 g, Coicis Semen (*Coix lacryma-jobi* var. *ma-yuen* (Rom.Caill.) Stapf) 50 g, Ganoderma (*Ganoderma sichuanense* J.D.Zhao & X.Q.Zhang) 50 g, Ligustri Lucidi Fructus (*Ligustrum lucidum* W.T.Aiton) 30 g, Lycii Fructus (*Lycium barbarum* L.) 30 g, Curcumae Radix (*Curcuma aromatica* Salisb.) 30 g, Massa Medicata Fermentata 20 g, Hordei Fructus Germinatus (*Hordeum vulgare* L.) 30 g
Modified YGJT	Zhao et al. (2021)	Pinelliae Rhizoma (*Pinellia ternata* (Thunb.) Makino) 15 g, Citri Reticulatae Pericarpium (*Citrus reticulata* Blanco) 10 g, Codonopsis Radix (*Codonopsis pilosula* (Franch.) Nannf.) 15 g, Poria (*Macrohyporia cocos* (Schwein.) I.Johans. & Ryvarden) 15 g, Atractylodis Macrocephalae Rhizoma (*Atractylodes macrocephala* Koidz.) 15 g, Glycyrrhizae Radix et Rhizoma (*Glycyrrhiza uralensis* Fisch. ex DC.) 6 g	Hedyotidis Herba (*Oldenlandia diffusa* (Willd.) Roxb.) 30 g, Scutellariae Barbatae Herba (*Scutellaria barbata* D.Don) 15 g, Solani Lyrati Herba (*Solanum lyratum* Thunb.) 15 g 1) Severe cough: add Eriobotryae Folium (*Eriobotrya japonica* (Thunb.) Lindl.) 15 g, Stemonae Radix (*Stemona sessilifolia* (Miq.) Miq.) 15 g 2) Loss of appetite: add Massa Medicata Fermentata 15 g, Oryzae Fructus Germinatus (*Oryza sativa* L.) 15 g 3) Chest tightness and pain: add Trichosanthis Fructus (*Trichosanthes kirilowii* Maxim.) 30 g, Allii Macrostemonis Bulbus (*Allium macrostemon* Bunge) 10 g, Inulae Flos (*Inula japonica* Thunb.) 10 g 4) Blood in phlegm: add Bletillae Rhizoma (*Bletilla striata* (Thunb.) Rchb.f.) 10 g, Imperatae Rhizoma (*Imperata cylindrica* (L.) Raeusch.) 15 g, Agrimoniae Herba (*Agrimonia pilosa* Ledeb.) 15 g 5) Pleural effusion: add Descurainiae Semen (*Descurainia sophia* (L.) Webb ex Prantl) 15 g, Solani Nigri Herba (*Solanum nigrum* L.) 30 g
Modified YGJT	Dong et al. (2018)	Pinelliae Rhizoma (*Pinellia ternata* (Thunb.) Makino) 15 g, Atractylodis Macrocephalae Rhizoma (*Atractylodes macrocephala* Koidz.) 15 g, Poria (*Macrohyporia cocos* (Schwein.) I.Johans. & Ryvarden) 15 g, Codonopsis Radix (*Codonopsis pilosula* (Franch.) Nannf.) 15 g, Citri Reticulatae Pericarpium (*Citrus reticulata* Blanco) 10 g, Glycyrrhizae Radix et Rhizoma (*Glycyrrhiza uralensis* Fisch. ex DC.) 6 g	Solani Lyrati Herba (*Solanum lyratum* Thunb.) 15 g, Scutellariae Barbatae Herba (*Scutellaria barbata* D.Don) 15 g, Hedyotidis Herba (*Oldenlandia diffusa* (Willd.) Roxb.) 30 g
Modified YGJT	Duan (2018)	Poria (*Macrohyporia cocos* (Schwein.) I.Johans. & Ryvarden) 9 g, Atractylodis Macrocephalae Rhizoma (*Atractylodes macrocephala* Koidz.) 9 g, Ginseng Radix et Rhizoma (*Panax ginseng* C.A.Mey.) 9 g, Glycyrrhizae Radix et Rhizoma (*Glycyrrhiza uralensis* Fisch. ex DC.) 10 g, Pinelliae Rhizoma (*Pinellia ternata* (Thunb.) Makino) 4.5 g, Citri Reticulatae Pericarpium (*Citrus reticulata* Blanco) 3 g	1) Severe cough: add Eriobotryae Folium (*Eriobotrya japonica* (Thunb.) Lindl.) 15 g, Stemonae Radix (*Stemona sessilifolia* (Miq.) Miq.) 15 g 2) Chest pain or chest discomfort: add Inulae Flos (*Inula japonica* Thunb.) 10 g, Trichosanthis Fructus (*Trichosanthes kirilowii* Maxim.) 30 g, Allii Macrostemonis Bulbus (*Allium macrostemon* Bunge) 10 g 3) Blood in sputum: add Agrimoniae Herba (*Agrimonia pilosa* Ledeb.) 15 g, Bletillae Rhizoma (*Bletilla striata* (Thunb.) Rchb.f.) 10 g, Imperatae Rhizoma (*Imperata cylindrica* (L.) Raeusch.) 15 g
YGJT	Harada et al. (2018)	Atractylodis Rhizoma (*Atractylodes lancea* (Thunb.) DC.), Ginseng Radix et Rhizoma (*Panax ginseng* C.A.Mey.), Pinelliae Rhizoma (*Pinellia ternata* (Thunb.) Makino), Poria (*Macrohyporia cocos* (Schwein.) I.Johans. & Ryvarden), Jujubae Fructus (*Ziziphus jujuba* Mill.), Citri Reticulatae Pericarpium (*Citrus reticulata* Blanco), Glycyrrhizae Radix et Rhizoma (*Glycyrrhiza uralensis* Fisch. ex DC.), Zingiberis Rhizoma Recens (*Zingiber officinale* Roscoe)	-
Modified YGJT	Li (2018)	Poria (*Macrohyporia cocos* (Schwein.) I.Johans. & Ryvarden) 15 g, Codonopsis Radix (*Codonopsis pilosula* (Franch.) Nannf.) 15 g, Pinelliae Rhizoma (*Pinellia ternata* (Thunb.) Makino) 15 g, Citri Reticulatae Pericarpium (*Citrus reticulata* Blanco) 10 g, Atractylodis Rhizoma (*Atractylodes lancea* (Thunb.) DC.) 10 g, Glycyrrhizae Radix et Rhizoma (*Glycyrrhiza uralensis* Fisch. ex DC.) 5 g	Trichosanthis Fructus (*Trichosanthes kirilowii* Maxim.) 10 g, Coicis Semen (*Coix lacryma-jobi* var. *ma-yuen* (Rom.Caill.) Stapf) 30 g 1) Vexation, phlegm heat, and poor sleep: add Aurantii Fructus Immaturus (*Citrus × aurantium* L.), Bambusae Caulis in Taenias (*Bambusa tuldoides* Munro) 2) Phlegm obstruction with shortness of breath: add Aurantii Fructus Immaturus (*Citrus × aurantium* L.), Arisaematis Rhizoma (*Arisaema erubescens* (Wall.) Schott) 3) Thick yellow phlegm: add Coptidis Rhizoma (*Coptis chinensis* Franch.), Trichosanthis Fructus (*Trichosanthes kirilowii* Maxim.) 4) Chest and flank distention and pain: add Curcumae Radix (*Curcuma aromatica* Salisb.), Aurantii Fructus Immaturus (*Citrus × aurantium* L.)
Modified YGJT	Liu et al. (2018)	Ginseng Radix et Rhizoma (*Panax ginseng* C.A.Mey.) 12 g, Atractylodis Macrocephalae Rhizoma (*Atractylodes macrocephala* Koidz.) 12 g, Poria (*Macrohyporia cocos* (Schwein.) I.Johans. & Ryvarden) 12 g, Glycyrrhizae Radix et Rhizoma (*Glycyrrhiza uralensis* Fisch. ex DC.) 6 g, Citri Reticulatae Pericarpium (*Citrus reticulata* Blanco) 6 g, Pinelliae Rhizoma (*Pinellia ternata* (Thunb.) Makino) 6 g	1) Severe cough: add Armeniacae Semen Amarum (*Prunus armeniaca* L.) 6 g, Platycodonis Radix (Platycodon grandiflorus (Jacq.) A.DC.) 6 g 2) Haemoptysis: add Agrimoniae Herba (*Agrimonia pilosa* Ledeb.) 6 g, Imperatae Rhizoma (*Imperata cylindrica* (L.) Raeusch.) 6 g 3) Severe chest pain: add Corydalis Rhizoma (*Corydalis yanhusuo* (Y.H.Chou & Chun C.Hsu) W.T.Wang ex Z.Y.Su & C.Y.Wu) 6 g, Paeoniae Radix Alba (*Paeonia lactiflora* Pall.)
Modified YGJT	Sun (2020)	Ginseng Radix et Rhizoma (*Panax ginseng* C.A.Mey.) 12 g, Atractylodis Macrocephalae Rhizoma (*Atractylodes macrocephala* Koidz.) 14 g, Poria (*Macrohyporia cocos* (Schwein.) I.Johans. & Ryvarden) 14 g, Glycyrrhizae Radix et Rhizoma (*Glycyrrhiza uralensis* Fisch. ex DC.) 6 g, Citri Reticulatae Pericarpium (*Citrus reticulata* Blanco) 6 g, Pinelliae Rhizoma (*Pinellia ternata* (Thunb.) Makino) 6 g	1) Cough: add Fritillariae Thunbergii Bulbus (*Fritillaria thunbergii* Miq.) 10 g, Armeniacae Semen Amarum (*Prunus armeniaca* L.) 10 g 2) Nausea and vomiting: add Inulae Flos (*Inula japonica* Thunb.) 10 g, Haematitum 15 g 3) Fatigue: add Ginseng Radix et Rhizoma (*Panax ginseng* C.A.Mey.) 15 g, Astragali Radix (*Astragalus mongholicus* Bunge) 15 g 4) Leukopenia: add Ligustri Lucidi Fructus (*Ligustrum lucidum* W.T.Aiton) 10 g, Hominis Placenta 15 g 5) Loose stools: add Amomi Fructus (*Wurfbainia villosa* (Lour.) Škorničk. & A.D.Poulsen) 5 g, Agastachis Herba (*Agastache rugosa* (Fisch. & C.A.Mey.) Kuntze) 10 g
Modified YGJT	Sun et al. (2021)	Glycyrrhizae Radix et Rhizoma (*Glycyrrhiza uralensis* Fisch. ex DC.) 6 g, Citri Reticulatae Pericarpium (*Citrus reticulata* Blanco) 6 g, Pinelliae Rhizoma (*Pinellia ternata* (Thunb.) Makino) 6 g, Ginseng Radix et Rhizoma (*Panax ginseng* C.A.Mey.) 12 g, Atractylodis Macrocephalae Rhizoma (*Atractylodes macrocephala* Koidz.), Poria (*Macrohyporia cocos* (Schwein.) I.Johans. & Ryvarden) 14 g	1) Cough: add Fritillariae Thunbergii Bulbus (*Fritillaria thunbergii* Miq.) 10 g, Armeniacae Semen Amarum (*Prunus armeniaca* L.) 10 g 2) Nausea and vomiting: add Inulae Flos (*Inula japonica* Thunb.) 10 g, Haematitum 15 g 3) Fatigue: add Ginseng Radix et Rhizoma (*Panax ginseng* C.A.Mey.) 15 g, Astragali Radix (*Astragalus mongholicus* Bunge) 15 g 4) Leukopenia: add Ligustri Lucidi Fructus (*Ligustrum lucidum* W.T.Aiton) 10 g, Hominis Placenta 15 g 5) Loose stools: add Amomi Fructus (*Wurfbainia villosa* (Lour.) Škorničk. & A.D.Poulsen) 5 g, Agastachis Herba (*Agastache rugosa* (Fisch. & C.A.Mey.) Kuntze) 10 g
Modified YGJT	Zhao et al. (2021)	Codonopsis Radix (*Codonopsis pilosula* (Franch.) Nannf.) 30 g, Atractylodis Macrocephalae Rhizoma (*Atractylodes macrocephala* Koidz.) 15 g, Poria (*Macrohyporia cocos* (Schwein.) I.Johans. & Ryvarden) 20 g, Glycyrrhizae Radix et Rhizoma (*Glycyrrhiza uralensis* Fisch. ex DC.) 10 g, Citri Reticulatae Pericarpium (*Citrus reticulata* Blanco) 5 g, Pinelliae Rhizoma (*Pinellia ternata* (Thunb.) Makino) 15 g	1) Severe phlegm-heat: add Arisaematis Rhizoma (*Arisaema erubescens* (Wall.) Schott) 15 g, Trichosanthis Fructus (*Trichosanthes kirilowii* Maxim.) 15 g 2) Excessive dampness: add Coicis Semen (*Coix lacryma-jobi* var. ma-yuen (Rom.Caill.) Stapf) 30 g, Polyporus (*Polyporus umbellatus* (Pers.) Fr.) 20 g 3) Phlegm and stasis accumulation: add Gecko (*Gekko gecko* (Linnaeus, 1758)) 6 g, Pheretima (*Amynthas aspergillum* (Perrier, 1872)) 3 g
JCYGJT	Cai et al. (2022)	Ginseng Radix et Rhizoma (*Panax ginseng* C.A.Mey.) 10 g, Atractylodis Macrocephalae Rhizoma (*Atractylodes macrocephala* Koidz.) 20 g, Poria (*Macrohyporia cocos* (Schwein.) I.Johans. & Ryvarden) 20 g, Pinelliae Rhizoma (Pinellia ternata (Thunb.) Makino) 15 g, Citri Reticulatae Pericarpium (*Citrus reticulata* Blanco) 15 g, Aurantii Fructus (*Citrus × aurantium* L.) 20 g, Aucklandiae Radix (*Dolomiaea costus* (Falc.) Kasana & A.K.Pandey) 15 g, Zingiberis Rhizoma Recens (*Zingiber officinale* Roscoe) 10 g, Jujubae Fructus (*Ziziphus jujuba* Mill.) 5 pieces, Glycyrrhizae Radix et Rhizoma (*Glycyrrhiza uralensis* Fisch. ex DC.) 6 g	-
Modified YGJT	Chen et al. (2022)	Ginseng Radix et Rhizoma (*Panax ginseng* C.A.Mey.) 10 g, Poria (*Macrohyporia cocos* (Schwein.) I.Johans. & Ryvarden) 15 g, Pinelliae Rhizoma (*Pinellia ternata* (Thunb.) Makino) 10 g, Atractylodis Macrocephalae Rhizoma (*Atractylodes macrocephala* Koidz.) 15 g, Citri Reticulatae Pericarpium (*Citrus reticulata* Blanco) 10 g, Atractylodis Macrocephalae Rhizoma (*Atractylodes macrocephala* Koidz.) 15 g, Glycyrrhizae Radix et Rhizoma (*Glycyrrhiza uralensis* Fisch. ex DC.) 3 g	Astragali Radix (*Astragalus mongholicus* Bunge) 20 g, Hedyotidis Herba (*Oldenlandia diffusa* (Willd.) Roxb.) 30 g, Ostreae Concha (*Magallana gigas* (Thunberg, 1793)) 15 g, Cremastrae Pseudobulbus (*Cremastra appendiculata* (D.Don) Makino) 30 g
Modified YGJT	Gao (2022)	Ginseng Radix et Rhizoma (*Panax ginseng* C.A.Mey.) 12 g, Pinelliae Rhizoma (*Pinellia ternata* (Thunb.) Makino) 6 g, Atractylodis Macrocephalae Rhizoma (*Atractylodes macrocephala* Koidz.)12 g, Citri Reticulatae Pericarpium (*Citrus reticulata* Blanco) 6 g, Poria (*Macrohyporia cocos* (Schwein.) I.Johans. & Ryvarden) 12 g, Glycyrrhizae Radix et Rhizoma (*Glycyrrhiza uralensis* Fisch. ex DC.) 6 g	1) Severe cough: add Armeniacae Semen Amarum (*Prunus armeniaca* L.) 6 g, Platycodonis Radix (*Platycodon grandiflorus* (Jacq.) A.DC.) 6 g 2) Hemoptysis: add Agrimoniae Herba (*Agrimonia pilosa* Ledeb.) 6 g, Imperatae Rhizoma (*Imperata cylindrica* (L.) Raeusch.) 6 g 3) Severe chest pain: add Corydalis Rhizoma (*Corydalis yanhusuo* (Y.H.Chou & Chun C.Hsu) W.T.Wang ex Z.Y.Su & C.Y.Wu) 6 g, Paeoniae Radix Alba (*Paeonia lactiflora* Pall.) 6 g
Modified HSYGJT	Guo et al. (2024)	Ginseng Radix et Rhizoma (*Panax ginseng* C.A.Mey.) 3 g, Atractylodis Macrocephalae Rhizoma (*Atractylodes macrocephala* Koidz.) 6 g, Poria (*Macrohyporia cocos* (Schwein.) I.Johans. & Ryvarden) 6 g, Glycyrrhizae Radix et Rhizoma (*Glycyrrhiza uralensis* Fisch. ex DC.) 2 g, Citri Reticulatae Pericarpium (*Citrus reticulata* Blanco) 2.5 g, Pinelliae Rhizoma (*Pinellia ternata* (Thunb.) Makino) 3 g, Amomi Fructus (*Wurfbainia villosa* (Lour.) Škorničk. & A.D.Poulsen) 2.5 g, Aucklandiae Radix (*Dolomiaea costus* (Falc.) Kasana & A.K.Pandey) 2 g	1) Severe abdominal pain: add Corydalis Rhizoma (*Corydalis yanhusuo* (Y.H.Chou & Chun C.Hsu) W.T.Wang ex Z.Y.Su & C.Y.Wu) 10 g 2) Severe cold-dampness: add Cinnamomi Cortex (*Cinnamomum cassia* (L.) J.Presl) 10 g 3) Acid reflux: add Sepiae Endoconcha [*Sepiella inermis* (Van Hasselt, 1835)] 10 g
Modified YGJT	Nie et al. (2022)	Poria (*Macrohyporia cocos* (Schwein.) I.Johans. & Ryvarden) 15 g, Atractylodis Macrocephalae Rhizoma (*Atractylodes macrocephala* Koidz.) 15 g, Ginseng Radix et Rhizoma (*Panax ginseng* C.A.Mey.) 15 g, Pinelliae Rhizoma (*Pinellia ternata* (Thunb.) Makino) 10 g, Citri Reticulatae Pericarpium (*Citrus reticulata* Blanco) 10 g, Glycyrrhizae Radix et Rhizoma (*Glycyrrhiza uralensis* Fisch. ex DC.) 6 g	1) Chest pain: add Paeoniae Radix Alba (*Paeonia lactiflora* Pall.) 20 g, Aurantii Fructus Immaturus (*Citrus × aurantium* L.) 10 g, Persicae Semen (*Prunus persica* (L.) Batsch) 10 g 2) Haemoptysis: add Imperatae Rhizoma (*Imperata cylindrica* (L.) Raeusch.) 20 g, Agrimoniae Herba (*Agrimonia pilosa* Ledeb.) 15 g 3) Yellow phlegm and cough: add Platycodonis Radix (*Platycodon grandiflorus* (Jacq.) A.DC.) 10 g, Scutellariae Radix (*Scutellaria baicalensis* Georgi) 10 g, Gardeniae Fructus (*Gardenia jasminoides* J.Ellis) 15 g
Modified JGYGJT	Wang et al. (2024)	Hordei Fructus Germinatus (*Hordeum vulgare* L.) 30 g, Codonopsis Radix (*Codonopsis pilosula* (Franch.) Nannf.) 20 g, Poria (*Macrohyporia cocos* (Schwein.) I.Johans. & Ryvarden) 20 g, Sepiae Endoconcha [*Sepiella inermis* (Van Hasselt, 1835)] 15 g, Fritillariae Thunbergii Bulbus (*Fritillaria thunbergii* Miq.) 15 g, Aurantii Fructus Immaturus (*Citrus × aurantium* L.) 10 g, Atractylodis Macrocephalae Rhizoma (*Atractylodes macrocephala* Koidz.) 10 g, Platycodonis Radix (*Platycodon grandiflorus* (Jacq.) A.DC.) 10 g, Pinelliae Rhizoma (*Pinellia ternata* (Thunb.) Makino) 10 g, Citri Reticulatae Pericarpium (*Citrus reticulata* Blanco) 10 g, Glycyrrhizae Radix et Rhizoma (*Glycyrrhiza uralensis* Fisch. ex DC.) 6 g	Astragali Radix (*Astragalus mongholicus* Bunge) 30 g, Galli Gigerii Endothelium Corneum (*Gallus gallus domesticus* (Linnaeus, 1758)) 15 g, Akebiae Fructus (*Akebia quinata* (Thunb. ex Houtt.) Decne.) 10 g, Saposhnikoviae Radix (*Saposhnikovia divaricata* (Turcz. ex Ledeb.) Schischk.) 6 g
Modified HSYGJT	Liu et al. (2023)	Codonopsis Radix (*Codonopsis pilosula* (Franch.) Nannf.) 30 g, Atractylodis Macrocephalae Rhizoma (*Atractylodes macrocephala* Koidz.) 15 g, Poria (*Macrohyporia cocos* (Schwein.) I.Johans. & Ryvarden) 10 g, Pinelliae Rhizoma (*Pinellia ternata* (Thunb.) Makino) 10 g, Citri Reticulatae Pericarpium (*Citrus reticulata* Blanco) 10 g, Aucklandiae Radix (*Dolomiaea costus* (Falc.) Kasana & A.K.Pandey) 10 g, Amomi Fructus (*Wurfbainia villosa* (Lour.) Škorničk. & A.D.Poulsen) 10 g, Glycyrrhizae Radix et Rhizoma (*Glycyrrhiza uralensis* Fisch. ex DC.) 6 g	Astragali Radix (*Astragalus mongholicus* Bunge) 30 g, Platycodonis Radix (*Platycodon grandiflorus* (Jacq.) A.DC.) 10 g, Armeniacae Semen Amarum (*Prunus armeniaca* L.) 10 g 1) Cough and sputum: add Mori Cortex (*Morus alba* L.), Fritillariae Cirrhosae Bulbus (*Fritillaria cirrhosa* D.Don), Coicis Semen (*Coix lacryma-jobi* var. *ma-yuen* (Rom.Caill.) Stapf), Raphani Semen (*Raphanus raphanistrum* subsp. *sativus* (L.) Domin) 2) Shortness of breath and chest tightness: add Trichosanthis Fructus (*Trichosanthes kirilowii* Maxim.), Aurantii Fructus Immaturus (*Citrus × aurantium* L.), Allii Macrostemonis Bulbus (*Allium macrostemon* Bunge) 3) Short breath and fatigue: add Gecko [*Gekko gecko* (Linnaeus, 1758)], Schisandrae Chinensis Fructus (*Schisandra chinensis* (Turcz.) Baill.), Lycii Fructus (*Lycium barbarum* L.) 4) Nausea and vomiting: add Aurantii Fructus Immaturus (*Citrus × aurantium* L.), Haematitum 5) Epigastric discomfort and acid reflux: add Bletillae Rhizoma (*Bletilla striata* (Thunb.) Rchb.f.), Sepiae Endoconcha (*Sepiella inermis* (Van Hasselt, 1835)) 10 g 6) Cold stomach and bitter taste: add Zingiberis Rhizoma Recens (*Zingiber officinale* Roscoe), Coptidis Rhizoma (*Coptis chinensis* Franch.) 7) Loose stools and bitter taste: add Euodiae Fructus (*Tetradium ruticarpum* (A.Juss.) T.G.Hartley), Coptidis Rhizoma (*Coptis chinensis* Franch.) 8) Dry stools: add Platycladi Semen (*Platycladus orientalis* (L.) Franco), Cannabis Fructus (*Cannabis sativa* L.), Asteris Radix et Rhizoma (*Aster tataricus* L.f.) 9) Dark tongue and choppy pulse: add Paeniae Radix Rubra (*Paeonia lactiflora* Pall.), Curcumae Rhizoma (*Curcuma phaeocaulis* Valeton) 10) Granulocytopenia: add Ligustri Lucidi Fructus (*Ligustrum lucidum* W.T.Aiton), Ecliptae Herba (*Eclipta prostrata* (L.) L.), Epimedii Folium (*Epimedium brevicornu* Maxim.), Cervi Cornus Colla (*Cervus elaphus* Linnaeus, 1758) 11) Severe anemia: add Asini Corii Colla (*Equus africanus asinus* Linnaeus, 1758), Angelicae Sinensis Radix (*Angelica sinensis* (Oliv.) Diels), Spatholobi Caulis (*Spatholobus suberectus* Dunn), Jujubae Fructus (*Ziziphus jujuba* Mill.) 12) Significant thrombocytopenia: add Corni Fructus (*Cornus officinalis* Siebold & Zucc.), Agrimoniae Herba (*Agrimonia pilosa* Ledeb.)
Modified YGJT	Tao and Liu (2023)	Ginseng Radix et Rhizoma (Panax ginseng C.A.Mey.) 15 g, Atractylodis Macrocephalae Rhizoma (Atractylodes macrocephala Koidz.) 10 g, Poria (Macrohyporia cocos (Schwein.) I.Johans. & Ryvarden) 15 g, Glycyrrhizae Radix et Rhizoma (Glycyrrhiza uralensis Fisch. ex DC.) 10 g, Citri Reticulatae Pericarpium (Citrus reticulata Blanco) 15 g, Pinelliae Rhizoma (Pinellia ternata (Thunb.) Makino) 10 g	Astragali Radix (*Astragalus mongholicus* Bunge) 20 g, Paeoniae Radix Alba (*Paeonia lactiflora* Pall.) 15 g, Lycii Fructus (*Lycium barbarum* L.) 15 g, Polygonati Rhizoma (*Polygonatum kingianum* Collett & Hemsl.) 15 g, Semen Cuscutae (*Cuscuta japonica* Choisy) 15 g, Adenophorae Radix (*Adenophora stricta* Miq.) 15 g, Ophiopogonis Radix (*Ophiopogon japonicus* (Thunb.) Ker Gawl.) 10 g
Modified YGJT	Wang et al. (2023)	Glycyrrhizae Radix et Rhizoma (*Glycyrrhiza uralensis* Fisch. ex DC.) 12 g, Pinelliae Rhizoma (*Pinellia ternata* (Thunb.) Makino) 12 g, Citri Reticulatae Pericarpium (*Citrus reticulata* Blanco) 15 g, Atractylodis Macrocephalae Rhizoma (*Atractylodes macrocephala* Koidz.) 15 g, Codonopsis Radix (*Codonopsis pilosula* (Franch.) Nannf.) 15 g, Poria (*Macrohyporia cocos* (Schwein.) I.Johans. & Ryvarden) 20 g, Jujubae Fructus (*Ziziphus jujuba* Mill.) 6 pieces, Zingiberis Rhizoma Recens (*Zingiber officinale* Roscoe) 10 g	Amomi Fructus (*Wurfbainia villosa* (Lour.) Škorničk. & A.D.Poulsen) 6 g, Aucklandiae Radix (*Dolomiaea costus* (Falc.) Kasana & A.K.Pandey) 10 g
JCYGJT	Bi et al. (2024)	Ginseng Radix et Rhizoma (*Panax ginseng* C.A.Mey.) 9 g, Atractylodis Macrocephalae Rhizoma (*Atractylodes macrocephala* Koidz.) 10 g, Poria (*Macrohyporia cocos* (Schwein.) I.Johans. & Ryvarden) 10 g, Pinelliae Rhizoma (*Pinellia ternata* (Thunb.) Makino) 10 g, Citri Reticulatae Pericarpium (*Citrus reticulata* Blanco) 10 g, Aurantii Fructus (*Citrus × aurantium* L.) 20 g, Aucklandiae Radix (*Dolomiaea costus* (Falc.) Kasana & A.K.Pandey) 15 g, Zingiberis Rhizoma Recens (*Zingiber officinale* Roscoe) 10 g, Jujubae Fructus (*Ziziphus jujuba* Mill.) 3 pieces, Glycyrrhizae Radix et Rhizoma (*Glycyrrhiza uralensis* Fisch. ex DC.) 6 g	-
Modified YGJT	Lin et al. (2024)	Atractylodis Macrocephalae Rhizoma (*Atractylodes macrocephala* Koidz.) 12 g, Poria (*Macrohyporia cocos* (Schwein.) I.Johans. & Ryvarden) 15 g, Codonopsis Radix (*Codonopsis pilosula* (Franch.) Nannf.) 15 g, Citri Reticulatae Pericarpium (*Citrus reticulata* Blanco) 9 g, Pinelliae Rhizoma (*Pinellia ternata* (Thunb.) Makino) 12 g	Selaginellae Herba (*Selaginella doederleinii* Hieron.) 30 g, Hedyotidis Herba (*Oldenlandia diffusa* (Willd.) Roxb.) 30 g, Salviae Chinensis Herba (*Salvia chinensis* Benth.) 30 g, Akebiae Fructus (*Akebia quinata* (Thunb. ex Houtt.) Decne.) 15 g, Prunellae Spica (*Prunella vulgaris* L.) 15 g, Arisaematis Rhizoma (*Arisaema erubescens* (Wall.) Schott) 15 g, Bufo (*Bufo gargarizans* Cantor, 1842) 9 g 1) Severe cough: add Eriobotryae Folium (*Eriobotrya japonica* (Thunb.) Lindl.) 15 g, Stemonae Radix (*Stemona sessilifolia* (Miq.) Miq.) 15 g 2) Loss of appetite: add Massa Medicata Fermentata 15 g, Oryzae Fructus Germinatus (*Oryza sativa* L.) 15 g 3) Nausea and vomiting: add Inulae Flos *(Inula japonica* Thunb.) 15 g, Haematitum 15 g 4) Chest tightness and pain: add Trichosanthis Fructus (*Trichosanthes kirilowii* Maxim.) 30 g, Allii Macrostemonis Bulbus (*Allium macrostemon* Bunge) 10 g, Inulae Flos (*Inula japonica* Thunb.) 10 g 5) Pleural effusion: add Descurainiae Semen (*Descurainia sophia* (L.) Webb ex Prantl) 15 g, Solani Nigri Herba (*Solanum nigrum* L.) 30 g 6) Leukopenia: add Hominis Placenta 10 g, Ligustri Lucidi Fructus (*Ligustrum lucidum* W.T.Aiton) 10 g

HSYGJT, Hyangsayukgunja-Tang; JCYGJT, Jichulyukgunja-Tang; JGYGJT, Jigilyukgunja-Tang; YGJT, Yukgunja-Tang.

The 31 RCTs enrolled a total of 2,496 patients: 1,256 in the treatment group and 1,240 in the control group. Harada et al. (2018) were analyzed as two separate entries Harada et al. (2018), as they reported outcomes separately for cisplatin- and carboplatin-based chemotherapy. The studies by Harada et al. (2018) were conducted in Japan, while all other studies were conducted in China, with publication years ranging from 2015 to 2024. Sample sizes ranged from 17 to 120 participants, and treatment durations varied from 4 to 10 months.

The modified YGJT formulations included YGJT, HSYGJT, JGYGJT, JCYGJT, and several variants of these formulations. In 29 studies, both groups received chemotherapy, while two studies used EGFR tyrosine kinase inhibitors (TKIs) in both groups (Zhao et al., 2021; Lin et al., 2024). Of the 31 studies, 29 compared modified YGJT combined with antitumor therapy against antitumor therapy alone. One study by Ye (2015) compared modified YGJT plus chemotherapy with omeprazole plus chemotherapy, while another by Chen et al. (2017) compared modified YGJT plus chemotherapy with placebo plus chemotherapy. These two studies were excluded from the meta-analysis due to the absence of comparable study designs. Accordingly, only studies directly comparing modified YGJT plus antitumor therapy with antitumor therapy alone were included to maintain methodological consistency and allow reliable meta-analysis.

Fifteen studies incorporated traditional East Asian pattern identification as a diagnostic criterion. Qi deficiency was reported across all studies. The distribution of specific syndromes was as follows:

• Qi deficiency of the spleen and lung: eight studies (Zhou et al., 2015; Li et al., 2017; Liu et al., 2018; Sun, 2020; Sun et al., 2021; Chen et al., 2022; Nie et al., 2022; Wang, 2022),
• Spleen deficiency with phlegm dampness: two studies (Zhao et al., 2021; Lin et al., 2024),
• Qi deficiency with phlegm dampness: two studies (Li, 2018; Bi et al., 2024),
• Spleen Qi deficiency: one study (Tao and Liu, 2023),
• Qi deficiency of the spleen and stomach: one study (Liu et al., 2023), and Qi deficiency (unspecified): one study (Cai et al., 2022).

### 3.3 Risk of bias

**FIGURE 2:**
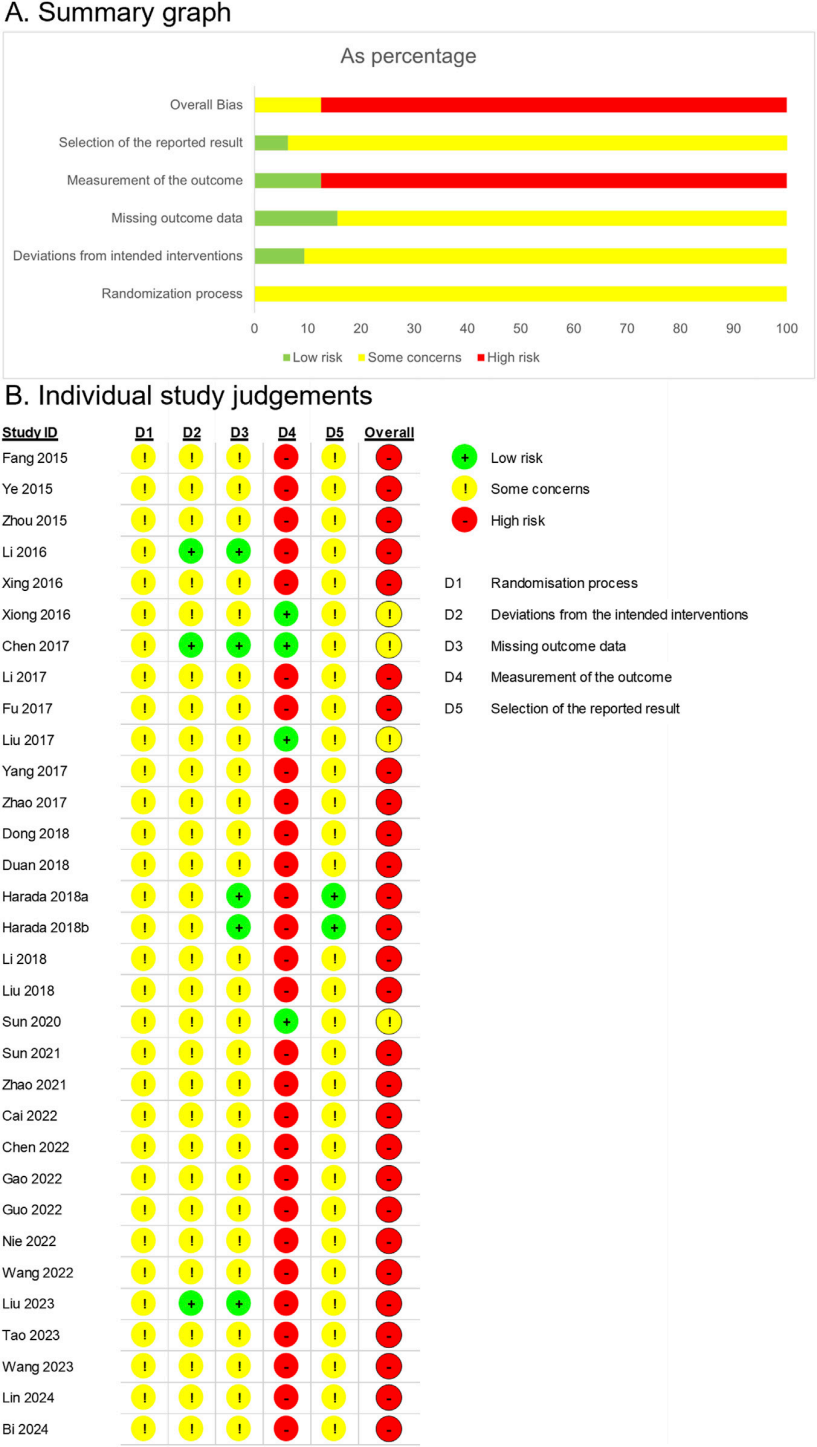
Risk of bias assessment based on the Cochrane Risk of Bias 2 tool. **(A)** Summary graph. **(B)** Individual study judgements.

All 31 RCTs were evaluated for the risk of bias (Figure 2).

In all the studies, the allocation sequence was either random or not reported, and no baseline differences were observed between the intervention groups. However, due to insufficient information on allocation concealment, the randomization process was rated as having some concerns.

For bias due to deviations from intended interventions, both participants and intervention providers were aware of the interventions assigned in the studies by Li et al. (2016) and Liu et al. (2023). However, the authors explicitly reported no dropouts, and all randomized participants were analyzed according to their allocated groups, suggesting that an intention-to-treat approach was likely followed. As there were no apparent deviations from the intended interventions, these studies were assessed to have a low risk of bias. Chen et al. (2017) employed a placebo, which likely ensured blinding of participants and intervention providers. The study explicitly stated that no dropouts occurred, and all randomized participants were included in the follow-up. Given these conditions, intention-to-treat analysis appeared to have been conducted, and the study was evaluated as having a low risk of bias. In contrast, Harada et al. (2018) reported one dropout outside the clinical trial context, where participants and intervention providers were aware of the assigned interventions. A per-protocol analysis was performed, and a single deviation was deemed unlikely to substantially affect the results. Consequently, this study was rated as having certain limitations. Zhao et al. (2021) conducted a study under conditions where both participants and intervention providers were aware of the assigned interventions. In the treatment group, there were instances of loss to follow-up, while in the control group, some participants discontinued the treatment, suggesting the possibility of deviation within the clinical trial context. Although no information was provided on whether these deviations influenced the outcomes, they appeared balanced between the two groups, and a per-protocol analysis, which excluded less than 5% of the participants, indicated a minimal likelihood of a significant impact on the results. Consequently, the study was rated as having concerns regarding risk of bias. In other studies, participants and intervention providers were aware of the assigned interventions; however, information on deviations and intention-to-treat analysis was not reported. All studies reported the results for all randomized participants; therefore, no substantial impact on the outcomes was evident. Thus, these studies were rated as having some concerns regarding the risk of bias.

Regarding missing outcome data, Li et al. (2016), Chen et al. (2017), Harada et al. (2018), and Liu et al. (2023) explicitly reported no dropouts, indicating a low risk of bias. Harada et al. (2018) reported a missing outcome due to disease progression before treatment initiation. Since this dropout rate was unlikely to be related to the true outcome value, the study was rated as having a low risk of bias. Zhao et al. (2021) reported dropouts in both groups, resulting in missing outcomes. However, the dropout rates and reasons were similar between the intervention and control groups, leading to some concerns. For the remaining studies, information on missing data was unavailable. Nonetheless, there were no indications of imbalanced dropout rates or differing reasons between the groups, and the results were reported for the full number of randomized participants. Consequently, these studies were rated as having some concerns regarding the risk of bias.

In Chen et al. (2017), the study was rated low risk due to the use of a placebo-controlled design. In Xiong and Xiong (2016), Liu et al. (2017), and Sun (2020), it is unlikely that the assessment of outcomes such as tumor response, immune markers, and tumor markers was influenced by awareness of the intervention received, resulting in a low rating of bias in the measurement of the outcome. In other studies, a modified YGJT was administered as an add-on therapy, and outcomes included subjective measures, leading to a high risk of bias in the outcome measurement.

Thirty studies were rated as having some concerns due to the lack of protocols. However, the study by Harada et al. (2018) was assessed as low risk, as it was conducted based on a predefined protocol.

### 3.4 Modified YGJT plus anti-tumor therapy versus anti-tumor therapy alone

#### 3.4.1 ORR (primary outcome)

**FIGURE 3:**
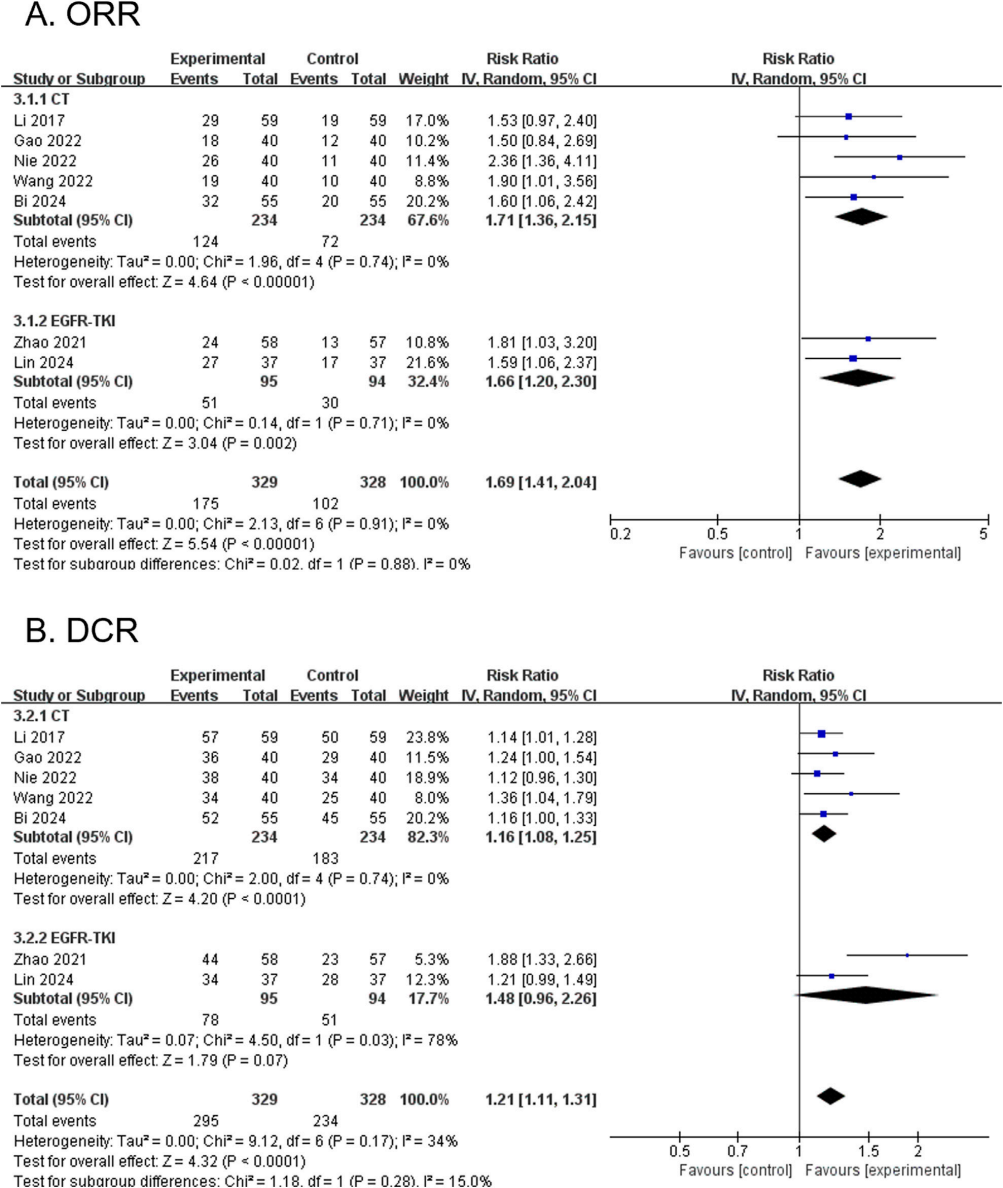
Forest plots comparing the effect of modified YGJT plus anti-tumor therapy versus anti-tumor therapy alone on tumor response outcomes. **(A)** Objective response rate (ORR). **(B)** Disease control rate (DCR). CI, confidence interval; CT, chemotherapy; EGFR-TKI, epidermal growth factor receptor tyrosine kinase inhibitor; IV, inverse variance; YGJT, Yukgunja-tang.

Seven RCTs, comprising 657 participants, reported the ORR. The modified YGJT plus anti-tumor therapy group showed a statistically significant improvement in ORR compared to the anti-tumor therapy alone group (RR 1.69, 95% CI 1.41 to 2.04, p < 0.00001) (Figure 3A). No significant heterogeneity was detected (p = 0.91, I^2^ = 0%). Subgroup analysis based on the type of anti-tumor therapy yielded results consistent with the main findings for both chemotherapy (RR 1.71, 95% CI 1.36 to 2.15; 5 RCTs; 468 participants) and EGFR-TKI (RR 1.66, 95% CI 1.20 to 2.30; 2 RCTs; 189 participants).

#### 3.4.2 DCR (primary outcome)

Seven RCTs, comprising 657 participants, reported the DCR. The modified YGJT intervention group demonstrated a statistically significant improvement in DCR compared to the control group (RR 1.21, 95% CI 1.11 to 1.31, p < 0.0001) (Figure 3B). Low heterogeneity was detected (p = 0.17, I^2^ = 34%). In the subgroup analysis by type of anti-tumor therapy, a significant effect was observed in the chemotherapy group (RR 1.16, 95% CI 1.08 to 1.25; 5 RCTs; 468 participants), whereas the EGFR-TKI group did not show a statistically significant result (RR 1.48, 95% CI 0.96 to 2.26; 2 RCTs; 189 participants).

#### 3.4.3 KPS score (dichotomous) (primary outcome)

**FIGURE 4:**
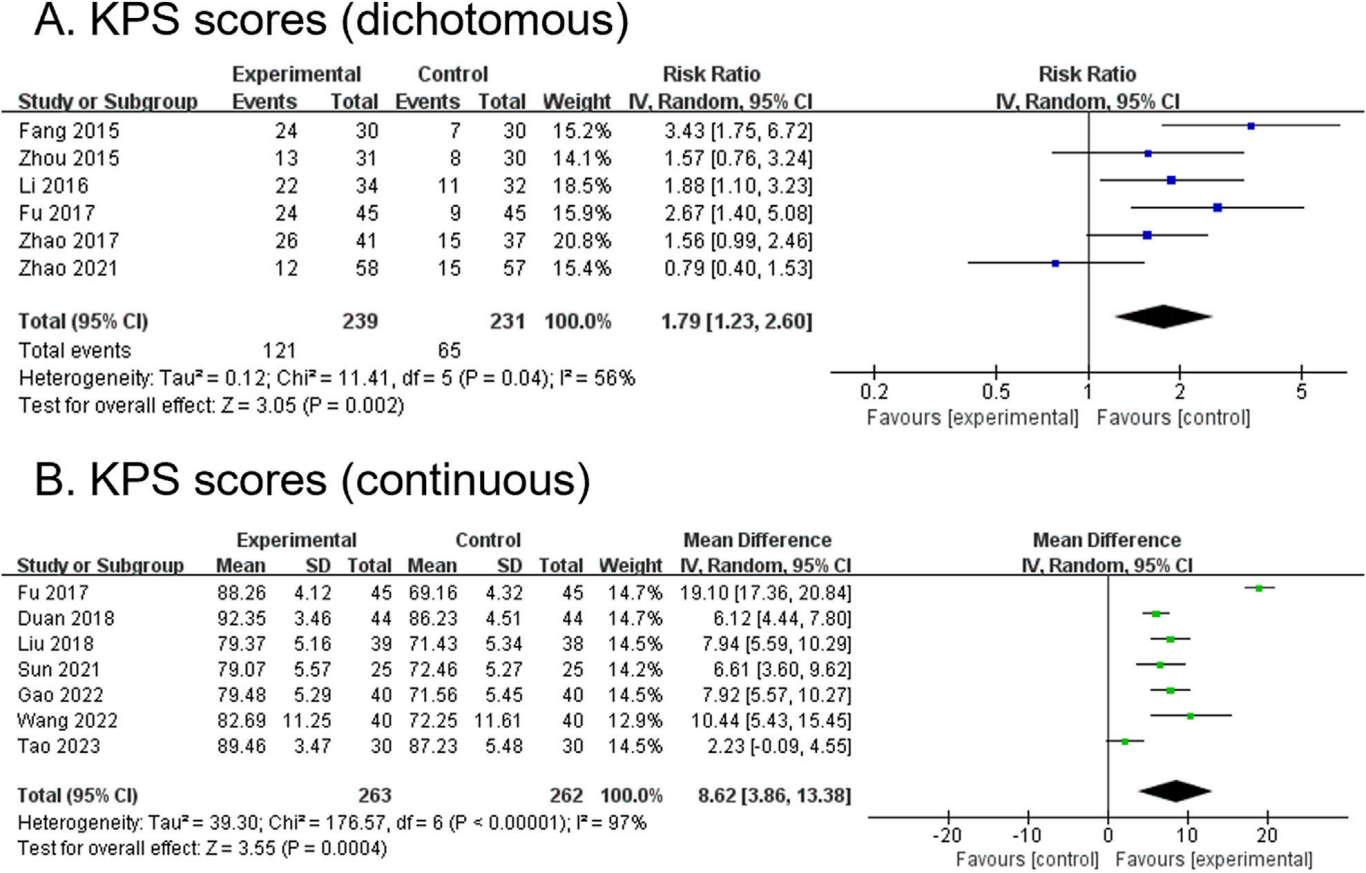
Forest plots comparing the effect of modified YGJT plus anti-tumor therapy versus anti-tumor therapy alone on Karnofsky Performance Status scores. **(A)** Dichotomous. **(B)** Continuous. CI, confidence interval; IV, inverse variance; SD, standard deviation; YGJT, Yukgunja-tang.

Six RCTs, comprising 470 participants, reported dichotomous outcomes for KPS scores. The modified YGJT intervention group showed a significant improvement in KPS scores compared to the control group (RR 1.79, 95% CI 1.23 to 2.60, p = 0.002) (Figure 4A). Moderate heterogeneity was observed (p = 0.04, I^2^ = 56%). Subgroup analysis was not performed due to fewer than two studies per subgroup.

#### 3.4.4 KPS score (continuous) (primary outcome)

Seven RCTs, comprising 525 participants, reported continuous outcomes for the KPS score. The modified YGJT intervention group demonstrated a significant increase in KPS scores compared to the control group (MD 8.62, 95% CI 3.86 to 13.38, p = 0.0004) (Figure 4B). Considerable heterogeneity was observed (p < 0.00001, I^2^ = 97%). Subgroup analysis could not be performed because fewer than two studies were available for each subgroup.

#### 3.4.5 Clinical symptom score (dichotomous) (secondary outcome)

**FIGURE 5:**
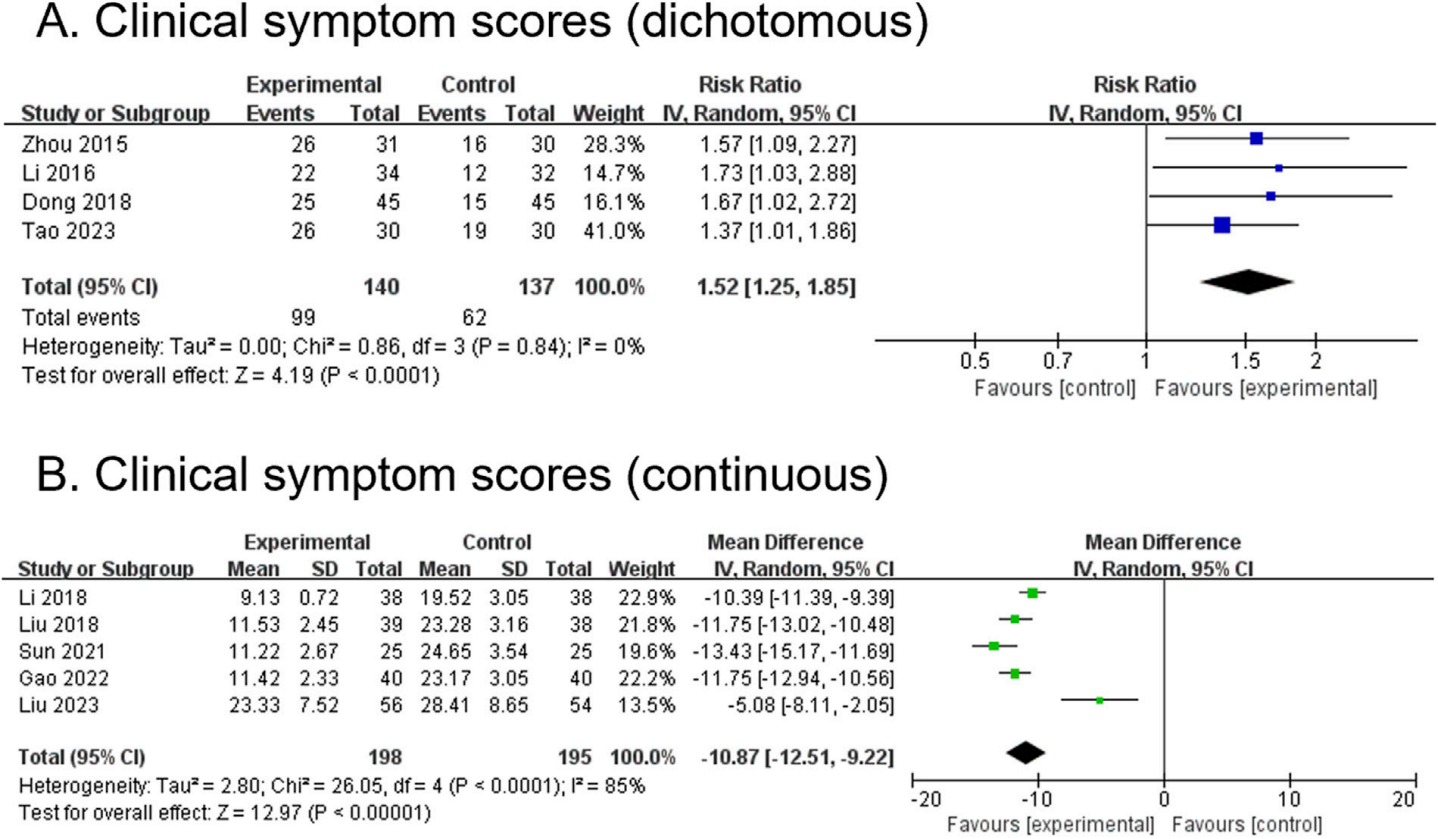
Forest plots comparing the effect of modified YGJT plus anti-tumor therapy versus anti-tumor therapy alone on clinical symptom scores. **(A)** Dichotomous. **(B)** Continuous. CI, confidence interval; IV, inverse variance; SD, standard deviation; YGJT, Yukgunja-tang.

Four RCTs, comprising 277 participants, reported dichotomous outcomes for clinical symptom scores. The modified YGJT group showed a significant improvement in clinical symptom scores compared to the control group (RR 1.52, 95% CI 1.25 to 1.85, p < 0.0001) (Figure 5A). No significant heterogeneity was observed (p = 0.84, I^2^ = 0%). Subgroup analysis was not conducted due to insufficient data across the groups.

#### 3.4.6 Clinical symptom score (continuous) (secondary outcome)

Five RCTs, comprising 393 participants, reported continuous outcomes for clinical symptom scores. The modified YGJT group showed a significant reduction in clinical symptoms compared to the control group (MD -10.87, 95% CI -12.51 to −9.22, p < 0.00001) (Figure 5B). Considerable heterogeneity was observed (p < 0.0001, I^2^ = 85%). Subgroup analysis was not performed due to insufficient data.

#### 3.4.7 CD3^+^ (secondary outcome)

**FIGURE 6:**
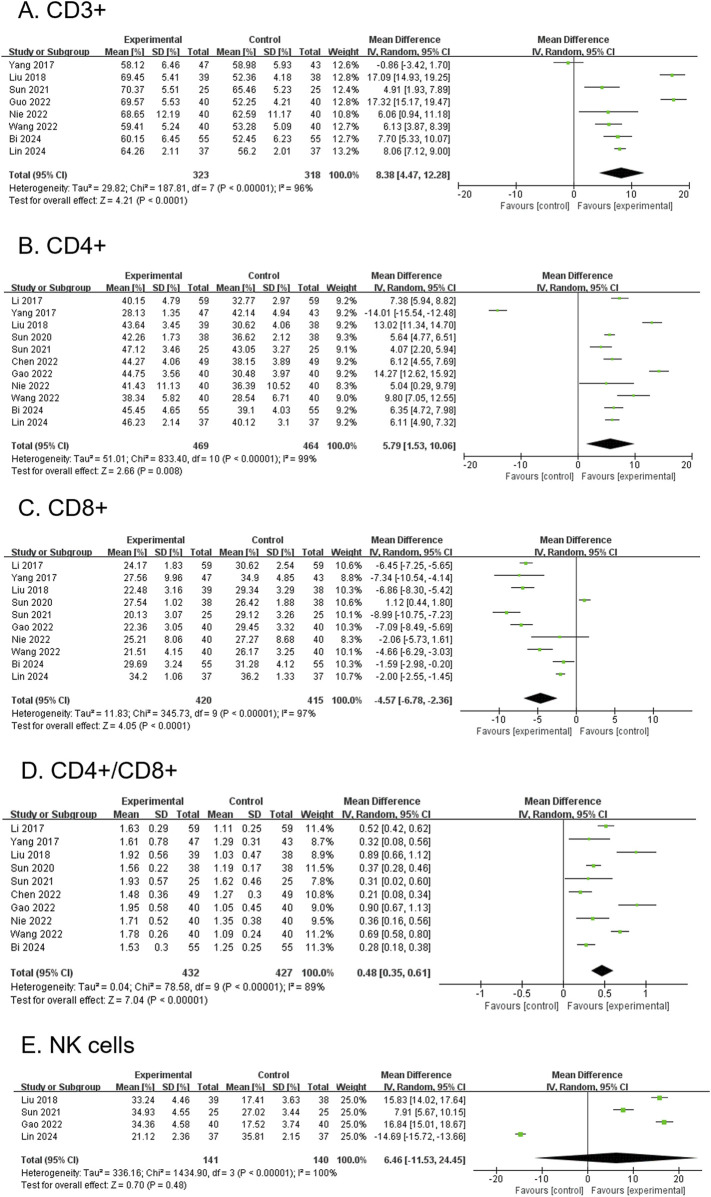
Forest plots comparing the effect of modified YGJT plus anti-tumor therapy versus anti-tumor therapy alone on immune function markers. **(A)** CD3^+^. **(B)** CD4^+^. **(C)** CD8^+^. **(D)** CD4+/CD8+. **(E)** NK Cells. CI, confidence interval; IV, inverse variance; SD, standard deviation; YGJT, Yukgunja-tang.

A total of 8 RCTs, comprising 641 participants, reported CD3^+^ levels. The modified YGJT group showed a significant increase in CD3^+^ levels compared to the control group (MD 8.38, 95% CI 4.47 to 12.28, p < 0.0001) (Figure 6A). Considerable heterogeneity was observed (p < 0.00001, I^2^ = 96%). Subgroup analysis was not feasible due to limited data within the relevant groups.

#### 3.4.8 CD4^+^ (secondary outcome)

A total of 11 RCTs, involving 933 participants, reported CD4^+^ levels. The modified YGJT group showed a significant increase in CD4^+^ levels compared to the control group (MD 5.79, 95% CI: 1.53–10.06, p = 0.008) (Figure 6B). Considerable heterogeneity was observed (p < 0.00001, I^2^ = 99%). The funnel plot suggested a potential publication bias; however, Egger’s test revealed no evidence of bias, with a regression intercept (bias) of 5.0421 (p = 0.8679) (Supplementary Figure S1A). Subgroup analysis was not feasible due to limited data across the relevant groups.

#### 3.4.9 CD8^+^ (secondary outcome)

Ten RCTs, involving 835 participants, reported CD8^+^ levels. The modified YGJT group showed a significant reduction in CD8^+^ levels compared to the control group (MD -4.57, 95% CI: −6.78 to −2.36, p < 0.0001) (Figure 6C). Considerable heterogeneity was observed (p < 0.00001, I^2^ = 97%). The funnel plot showed no clear evidence of publication bias, and Egger’s test did not suggest significant publication bias or a small-study effect (Egger’s regression intercept = −3.2948, p = 0.4843) (Supplementary Figure S1B). Subgroup analysis was not possible due to insufficient data across the relevant groups.

#### 3.4.10 CD4+/CD8+ (secondary outcome)

Ten RCTs, involving 859 participants, reported CD4+/CD8+ ratios. The modified YGJT group showed a significant increase in the CD4+/CD8+ ratio compared to the control group (MD 0.48, 95% CI: 0.35 to 0.61, p < 0.00001) (Figure 6D). Considerable heterogeneity was observed (p < 0.00001, I^2^ = 89%). The funnel plot showed no clear evidence of publication bias, and Egger’s test did not detect significant publication bias or a small-study effect (Egger’s regression intercept = 0.3616, p = 0.5395) (Supplementary Figure S1C). A subgroup analysis was not performed due to insufficient data across the study groups.

#### 3.4.11 NK cell (secondary outcome)

Four RCTs, involving 281 participants, reported NK cell levels. The modified YGJT group exhibited an increase in NK cell levels compared to the control group; however, this was not statistically significant (MD 6.46, 95% CI: −11.53 to 24.45, p = 0.48) (Figure 6E). Considerable heterogeneity was observed (p < 0.00001, I^2^ = 100%). Subgroup analysis was not feasible due to limited data.

#### 3.4.12 CEA (secondary outcome)

**FIGURE 7:**
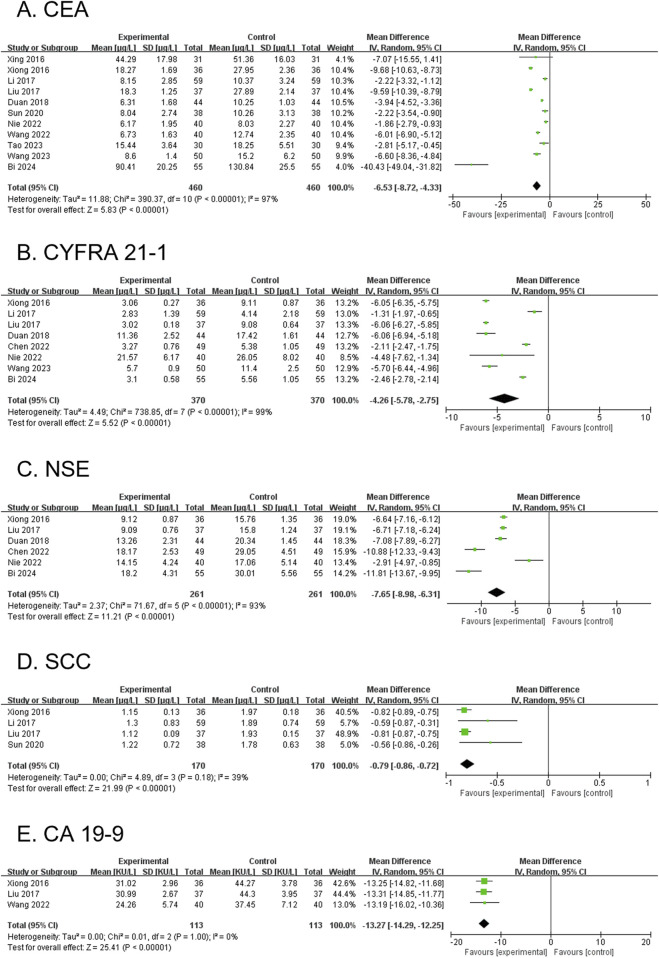
Forest plots comparing the effect of modified YGJT plus anti-tumor therapy versus anti-tumor therapy alone on tumor markers. **(A)** CEA. **(B)** CYFRA 21-1. **(C)** NSE. **(D)** SCC. **(E)** CA 19-9. CI, confidence interval; IV, inverse variance; SD, standard deviation; YGJT, Yukgunja-tang.

Eleven RCTs, involving 920 participants, reported CEA levels. The modified YGJT group showed a significant reduction in CEA levels compared to the control group (MD -6.53, 95% CI: −8.72 to −4.33, p < 0.00001) (Figure 7A). Considerable heterogeneity was observed (p < 0.00001, I^2^ = 97%). The funnel plot suggested potential publication bias or a small-study effect, as confirmed by Egger’s test (Egger’s regression intercept (bias) = −2.2596, p = 0.0025) (Supplementary Figure S1D). Subgroup analysis was not conducted due to limited subgroup data.

#### 3.4.13 CYFRA 21-1 (secondary outcome)

Eight RCTs, involving 740 participants, reported CYFRA 21-1 levels. The modified YGJT group showed a significant reduction in CYFRA 21-1 levels compared to the control group (MD -4.26, 95% CI: −5.78 to −2.75, p < 0.00001) (Figure 7B). Considerable heterogeneity was observed (p < 0.00001, I^2^ = 99%). Planned subgroup analysis could not be performed due to limited subgroup data.

#### 3.4.14 NSE (secondary outcome)

Six RCTs, involving 522 participants, reported NSE levels. The modified YGJT group exhibited a significant reduction in NSE levels compared to the control group (MD -7.65, 95% CI: −8.98 to −6.31, p < 0.00001) (Figure 7C). Substantial heterogeneity was observed (P < 0.00001, I^2^ = 93%). A subgroup analysis was not conducted due to insufficient data within the subgroups.

#### 3.4.15 SCC (secondary outcome)

Four RCTs, involving 340 participants, reported SCC antigen levels. The modified YGJT group showed a significant reduction in SCC levels compared to the control group (MD -0.79, 95% CI: −0.86 to −0.72, p < 0.00001) (Figure 7D). Moderate heterogeneity was observed (p = 0.18, I^2^ = 39%). A subgroup analysis was not performed due to data limitations.

#### 3.4.16 CA19-9 (secondary outcome)

Three RCTs, involving 226 participants, reported CA19-9 levels. The modified YGJT group showed a significant reduction in CA19-9 levels compared to the control group (MD -13.27, 95% CI: −14.29 to −12.25, p < 0.00001) (Figure 7E). No significant heterogeneity was observed (p = 1.00, I^2^ = 0%). A subgroup analysis was not performed due to limited data across groups.

#### 3.4.17 Myelosuppression (secondary outcome)

**FIGURE 8:**
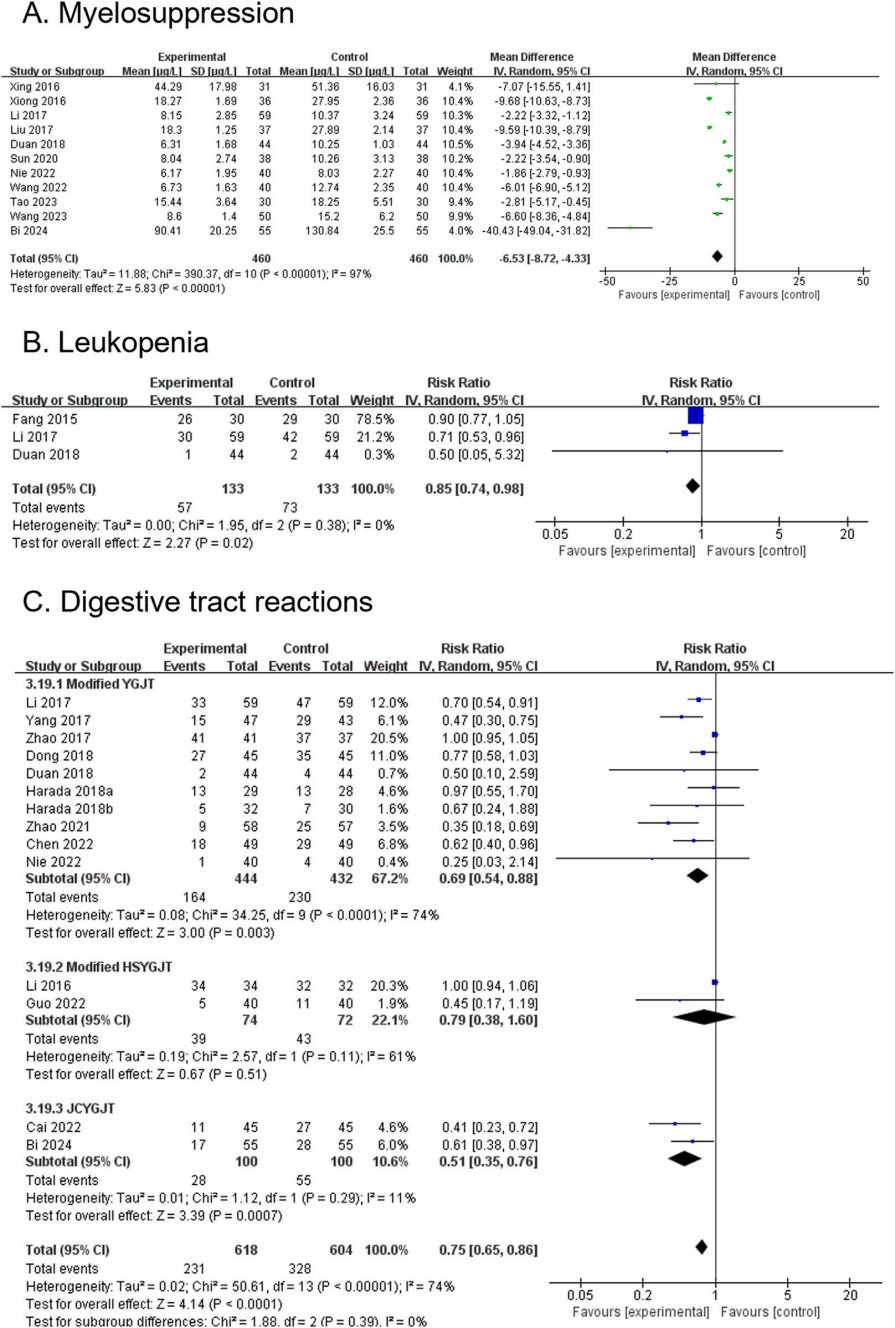
Forest plots comparing the effect of modified YGJT plus anti-tumor therapy versus anti-tumor therapy alone on adverse events. **(A)** Myelosuppression. **(B)** Leukopenia. **(C)** Digestive tract reactions. CI, confidence interval; IV, inverse variance; YGJT, Yukgunja-tang.

Seven RCTs, involving 634 participants, reported myelosuppression. The modified YGJT group showed a marginally significant reduction in myelosuppression compared to the control group (RR 0.63, 95% CI: 0.43–0.92, p = 0.02) (Figure 8A). Substantial heterogeneity was observed (p = 0.003, I^2^ = 69%). A subgroup analysis was not feasible due to limited data across groups.

#### 3.4.18 Leukopenia (secondary outcome)

Six RCTs, involving 266 participants, reported leukopenia. The modified YGJT group showed a significant reduction in leukopenia compared to the control group (RR 0.85, 95% CI: 0.74–0.98, p = 0.02) (Figure 8B). No significant heterogeneity was observed (p = 0.38, I^2^ = 0%). A subgroup analysis was not performed due to insufficient data.

#### 3.4.19 Digestive tract reaction (secondary outcome)

Fourteen RCTs, involving 1,222 participants, reported digestive tract reactions. The modified YGJT group showed a significant reduction in digestive tract reactions compared to the control group (RR 0.75, 95% CI: 0.65–0.86, p < 0.0001) (Figure 8C). Substantial heterogeneity was observed (P < 0.00001, I^2^ = 74%). Subgroup analysis based on the type of YGJT showed results consistent with the main findings for modified YGJT (RR 0.69, 95% CI: 0.54–0.88; 10 RCTs; 876 participants) and JCYGJT (RR 0.51, 95% CI: 0.35–0.76; 2 RCTs; 200 participants). However, the effect in the modified HSYGJT group was no longer significant (RR 0.79, 95% CI: 0.38–1.60; 2 RCTs; 146 participants). The funnel plot suggested potential publication bias, and Egger’s test indicated a significant publication bias or small study effect (Egger’s regression intercept (bias) 0.0512, p < 0.0001) (Supplementary Figure S1E).

### 3.5 Modified YGJT plus anti-tumor therapy versus placebo plus anti-tumor therapy

Chen et al. (2017) conducted a study comparing YGJT dry powder extract with a dry powder placebo in patients with advanced NSCLC receiving gemcitabine plus cisplatin therapy. After a median treatment duration of 16 weeks, no significant differences were observed between the YGJT and placebo groups in tumor response or adverse events.

### 3.6 Modified YGJT plus anti-tumor therapy versus active control plus anti-tumor therapy

Ye (2015) compared modified YGJT with omeprazole in patients with lung cancer undergoing chemotherapy. The modified YGJT group showed a significantly greater improvement in clinical symptom scores compared to the omeprazole group (p < 0.05).

### 3.7 Sensitivity analysis

For advanced-stage lung cancer, the sensitivity analysis demonstrated the same ORR (RR 1.69, 95% CI:1.41–2.04) and DCR (RR 1.21, 95% CI: 1.11–1.31) as the primary analysis, as the studies included in both analyses were identical. Improvements were confirmed in KPS score (RR 1.43, 95% CI: 1.01–2.03; MD 7.79, 95% CI: 6.05–9.52) and clinical symptom score (RR 1.62, 95% CI: 1.20–2.19; MD -12.47, 95% CI: −14.09 to −10.84). Immune function markers, including CD3^+^ (MD 7.11, 95% CI: 5.90–8.32), CD4^+^ (MD 7.25, 95% CI: 5.36–9.13), and CD4+/CD8+ ratio (MD 0.45, 95% CI: 0.31–0.59), continued to show benefits, with a reduction in CD8^+^ levels (MD -3.97, 95% CI: −6.41 to −1.54). Among tumor markers, CEA (MD -7.29, 95% CI: −10.38 to −4.20), CYFRA 21-1 (MD -3.64, 95% CI: −5.59 to −1.70), NSE (MD -8.10, 95% CI: −11.27 to −4.93), SCC (MD -0.70, 95% CI: −0.88 to −0.52), and CA19-9 (MD -13.28, 95% CI: −14.63 to −11.93) all showed consistent reductions in sensitivity analysis. The analysis also found reductions in myelosuppression (RR 0.63, 95% CI: 0.33–1.21), leukopenia (RR 0.71, 95% CI: 0.53–0.96), and digestive tract reactions (RR 0.79, 95% CI: 0.68–0.92). In contrast, the effect on NK cells (MD 3.34, 95% CI: −18.12–24.80) was less conclusive due to wide confidence intervals.

In studies that incorporated pattern differentiation as part of the diagnostic criteria, the results demonstrated a slightly higher ORR (RR 1.72, 95% CI 1.41–2.09) and a comparable DCR (RR 1.21, 95% CI 1.10–1.33) compared to the primary analysis and the sensitivity analysis for advanced cancer. Improvements in the KPS score were maintained; however, the effect weakened in the continuous analysis (MD 6.48, 95% CI 3.10–9.86) and became statistically non-significant in the dichotomous analysis (RR 1.10, 95% CI 0.56–2.16). Similarly, clinical symptom scores retained their directionality but showed a reduced effect size (RR 1.45, 95% CI 1.14 to 1.83; MD -10.51, 95% CI -12.73 to −8.29). Immune function markers, including CD3^+^ (MD 8.46, 95% CI 5.08–11.84), CD4^+^ (MD 7.10, 95% CI 5.46–8.73), and the CD4+/CD8+ ratio (MD 0.45, 95% CI 0.31–0.59), continued to demonstrate benefits. CD8^+^ levels still decreased but with a reduced magnitude (MD -3.94, 95% CI -6.36 to −1.53), while NK cell levels showed a significant reduction (MD -5.17, 95% CI -6.00 to −4.34). For tumor markers, such as CEA (MD -5.56, 95% CI -8.32 to −2.80), CYFRA 21-1 (MD -2.10, 95% CI -2.65 to −1.54), NSE (MD -8.57, 95% CI -13.61 to −3.52), SCC (MD -0.58, 95% CI -0.78 to −0.37), and CA19-9 (MD -13.19, 95% CI -16.02 to −10.36), the results remained consistent or showed a slight attenuation compared to the primary analysis and the advanced cancer sensitivity analysis. Regarding adverse events, studies incorporating pattern differentiation demonstrated greater reductions in myelosuppression (RR 0.45, 95% CI 0.27–0.74), leukopenia (RR 0.71, 95% CI 0.53–0.96), and digestive tract reactions (RR 0.58, 95% CI 0.46–0.72). A comprehensive summary comparing the results of these sensitivity analyses with the main analysis for all outcomes is presented in Table 3.

**TABLE 3:** Summary of results from the main meta-analysis and sensitivity analyses.

Outcome	Effect measure (RR/MD)	Main (95% CI)	Advanced cancer (95% CI)	Pattern identification (95% CI)
ORR	RR	1.69 (1.41–2.04)	1.69 (1.41–2.04)	1.72 (1.41–2.19)
DCR	RR	1.21 (1.11–1.31)	1.21 (1.11–1.31)	1.21 (1.10–1.33)
KPS Score (Dichotomous)	RR	1.79 (1.23–2.60)	1.43 (1.01–2.03)	1.10 (0.56–2.16)
KPS Score (Continuous)	MD	8.62 (3.86–13.38)	7.79 (6.05–9.52)	6.48 (3.10–9.86)
Clinical Symptom Score (Dichotomous)	RR	1.52 (1.25–1.85)	1.62 (1.20–2.19)	1.45 (1.14–1.83)
Clinical Symptom Score (Continuous)	MD	−10.87 (−12.51 to −9.22)	−12.47 (−14.09 to −10.84)	−10.51 (−12.73 to −8.29)
CD3^+^	MD	8.38 (4.47–12.28)	7.11 (5.90–8.32)	8.46 (5.08–11.84)
CD4^+^	MD	5.79 (1.53–10.06)	7.25 (5.36–9.13)	7.10 (5.46–8.73)
CD8^+^	MD	−4.57 (−6.78 to −2.36)	−3.97 (−6.41 to −1.54)	−3.94 (−6.36 to −1.53)
CD4+/CD8+ Ratio	MD	0.48 (0.35–0.61)	0.45 (0.31–0.59)	0.45 (0.31–0.59)
NK Cell	MD	6.46 (−11.53–24.45)	3.34 (−18.12–24.80)	−5.17 (−6.00 to −4.34)
CEA	MD	−6.53 (−8.72 to −4.33)	−7.29 (−10.38 to −4.20)	−5.56 (−8.32 to −2.80)
CYFRA 21-1	MD	−4.26 (−5.78 to −2.75)	−3.64 (−5.59 to −1.70)	−2.10 (−2.65 to −1.54)
NSE	MD	−7.65 (−8.98 to −6.31)	−8.10 (−11.27 to −4.93)	−8.57 (−13.61 to −3.52)
SCC	MD	−0.79 (−0.86 to −0.72)	−0.70 (−0.88 to −0.52)	−0.58 (−0.78 to −0.37)
CA19-9	MD	−13.27 (−14.29 to −12.25)	−13.28 (−14.63 to −11.93)	−13.19 (−16.02 to −10.36)
Myelosuppression	RR	0.63 (0.43–0.92)	0.63 (0.33–1.21)	0.45 (0.27–0.74)
Leukopenia	RR	0.85 (0.74–0.98)	0.71 (0.53–0.96)	0.71 (0.53–0.96)
Digestive Tract Reaction	RR	0.75 (0.65–0.86)	0.79 (0.68–0.92)	0.58 (0.46–0.72)

CA19-9, carbohydrate antigen 19-9; CEA, carcinoembryonic antigen; CI, confidence interval; CYFRA, 21-1, cytokeratin 19 fragment 21-1; DCR, disease control rate; KPS, karnofsky performance status; MD, mean difference; NK, natural killer; NSE, neuron-specific enolase; ORR, objective response rate; RR, risk ratio; SCC, squamous cell carcinoma antigen.

### 3.8 Certainty of evidence

The certainty of the evidence was assessed using the GRADE approach, with detailed results presented in Table 4. The outcomes assessed included ORR, DCR, and clinical symptom score (dichotomous), rated as high; SCC and CA19-9, rated as moderate; NK cell, CEA, and digestive tract reactions, rated as very low; and the remaining outcomes, rated as low.

**TABLE 4:** GRADE summary of findings for the comparison of modified YGJT plus anti-tumor therapy versus anti-tumor therapy alone.

Modified YGJT plus anti-tumor therapy compared to anti-tumor therapy alone for lung cancer					
Outcomes	Anticipated absolute effects* (95% CI)		Relative effect (95% CI)	No of participants (studies)	Certainty of the evidence (GRADE)
	Risk with anti-tumor therapy alone	Risk with modified YGJT plus anti-tumor therapy			
ORR	311 per 1,000	**526 per 1,000** (438–634)	**RR 1.69** (1.41–2.04)	657 (7 RCTs)	⊕⊕⊕○ Moderate^a^
DCR	713 per 1,000	**863 per 1,000** (792–935)	**RR 1.21** (1.11–1.31)	657 (7 RCTs)	⊕⊕⊕○ Moderate^a^
KPS Score (Dichotomous)	281 per 1,000	**504 per 1,000** (346–732)	**RR 1.79** (1.23–2.60)	470 (6 RCTs)	⊕⊕○○ Low^a^ ^,^ ^b^
KPS Score (Continuous)		MD **8.62 higher** (3.86 higher to 13.38 higher)	—	525 (7 RCTs)	⊕⊕○○ Low^a^ ^,^ ^b^
Clinical Symptom Score (Dichotomous)	453 per 1,000	**688 per 1,000** (566–837)	**RR 1.52** (1.25–1.85)	277 (4 RCTs)	⊕⊕⊕○ Moderate^a^
Clinical Symptom Score (Continuous)		MD **10.87 lower** (12.51 lower to 9.22 lower)	—	393 (5 RCTs)	⊕⊕○○ Low^a^ ^,^ ^b^
CD3^+^		MD **8.38 higher** (4.47 higher to 12.28 higher)	—	641 (8 studies)	⊕⊕○○ Low^a^ ^,^ ^b^
CD4^+^		MD **5.79 higher** (1.53 higher to 10.06 higher)	—	933 (11 RCTs)	⊕⊕○○ Low^a^ ^,b^
CD8^+^		MD **4.57 lower** (6.78 lower to 2.36 lower)	—	835 (10 RCTs)	⊕⊕○○ Low^a^ ^,b^
CD4+/CD8+		MD **0.48 higher** (0.35 higher to 0.61 higher)	—	859 (10 RCTs)	⊕⊕○○ Low^a^ ^,^ ^b^
NK Cell		MD **6.46 higher** (11.53 lower to 24.45 higher)	—	281 (4 RCTs)	⊕○○○ Very low^a^ ^,^ ^b^ ^,^ ^c^
CEA		MD **6.53 lower** (8.72 lower to 4.33 lower)	—	920 (11 RCTs)	⊕○○○ Very low^a^ ^,^ ^b^ ^,^ ^e^
CYFRA 21-1		MD **4.26 lower** (5.78 lower to 2.75 lower)	—	740 (8 RCTs)	⊕⊕○○ Low^a^ ^,^ ^b^
NSE		MD **7.65 lower** (8.98 lower to 6.31 lower)	—	522 (6 RCTs)	⊕⊕○○ Low^a^ ^,^ ^b^
SCC		MD **0.79 lower** (0.86 lower to 0.72 lower)	—	340 (4 RCTs)	⊕⊕⊕○ Moderate^a^
CA19-9		MD **13.27 lower** (14.29 lower to 12.25 lower)	—	226 (3 RCTs)	⊕⊕⊕○ Moderate^a^
Myelosuppression	415 per 1,000	**262 per 1,000** (179–382)	**RR 0.63** (0.43–0.92)	634 (7 RCTs)	⊕⊕○○ Low^a^ ^,^ ^b^
Leukopenia	549 per 1,000	**467 per 1,000** (406–538)	**RR 0.85** (0.74–0.98)	266 (3 RCTs)	⊕⊕○○ Low^a^ ^,^ ^d^
Digestive Tract Reaction	543 per 1,000	**407 per 1,000** (353–467)	**RR 0.75** (0.65–0.86)	1222 (14 RCTs)	⊕○○○ Very low^a^ ^,^ ^b^ ^,^ ^e^

Explanations.

^a^ Downgraded due to study limitations (concerns or high risk of bias).

^b^ Downgraded due to inconsistency across studies.

^c^ Downgraded due to imprecision, as the confidence intervals include no effect.

^d^ Downgraded due to imprecision, as the optimal information size was not achieved.

^e^ Downgraded due to concerns about potential publication bias.

Bold values indicate the primary summary effect measure for each outcome.

All outcomes were downgraded due to the risk of bias. The KPS score, clinical symptom score (continuous), CD3^+^, CD4^+^, CD8^+^, CD4+/CD8+ ratio, NK cells, CEA, CYFRA 21-1, NSE, myelosuppression, and digestive tract reaction were further downgraded by one level due to inter-study inconsistencies. NK cell levels were additionally downgraded by one level for imprecision because the confidence interval included the null effect (0). Leukopenia was downgraded by one level for imprecision because the optimal information size criterion was not met. The digestive tract reaction was downgraded by one level due to indications of potential publication bias.

## 4 Discussion

This systematic review included 31 RCTs with 2,496 participants who met the inclusion criteria. Modified YGJT combined with antitumor therapy improved both ORR and DCR compared to antitumor therapy alone. Furthermore, YGJT significantly enhanced patients’ quality of life and immune function, while reducing tumor markers and treatment-related adverse events.

Lung cancer has the highest incidence and mortality of all cancers worldwide, with an estimated annual incidence of 2.48 million cases and 1.82 million deaths (Bray et al., 2024). Significant advancements have been made in standard treatment modalities, surgery, chemotherapy, radiotherapy, targeted therapy, and immune checkpoint inhibition, along with recent progress in nano-drug delivery systems, molecular targeted therapies, photothermal approaches, and immunotherapy, all contributing to incremental improvements in survival rates (Thai et al., 2021; Li et al., 2023). Nevertheless, the 5-year survival rate remains alarmingly low, highlighting persistent challenges in disease management (Li et al., 2023). As a result, the therapeutic potential of traditional herbal medicine is increasingly recognized as a promising complementary strategy to enhance efficacy and alleviate patient burden.

In traditional East Asian medicine, the pathology of lung cancer is attributed to root deficiency with excess tip, where an underlying Qi deficiency in the spleen and lung leads to the formation of byproducts, such as blood stasis or phlegm dampness (Liu et al., 2018). This imbalance is often exacerbated during anti-tumor therapy, as patients frequently experience physical weakness manifesting as symptoms of Qi deficiency and phlegm dampness, including nausea, vomiting, fatigue, and malaise. These symptoms ultimately reduce treatment adherence and patient quality of life. To address these issues, traditional East Asian medicine applies the primary principles of “strengthening the body” and “eliminating evil,” which refer to enhancing immune defenses against tumors and inhibiting tumor growth, proliferation, and metastasis in terms of Western medicine (Li et al., 2021).

YGJT is a representative prescription for the treatment of spleen Qi deficiency and phlegm dampness. YGJT exerts effects such as tonifying the spleen and lung, benefiting Qi and nourishing blood, and expelling dampness: Ginseng Radix et Rhizoma strengthens the spleen and benefits the lung, replenishes Yuan-primordial Qi, nourishes blood, and generates fluids; Poria strengthens the spleen, calms the mind, and drains dampness; Atractylodis Macrocephalae Rhizoma tonifies the spleen and benefits the lung, dries dampness, and promotes urination; Citri Reticulatae Pericarpium harmonizes the middle jiao, circulates Qi, disperses nodules, and dries dampness; Pinelliae Rhizoma downregulates Qi, resolves masses, transforms phlegm, and dries dampness; and Glycyrrhizae Radix et Rhizoma tonifies the spleen, nourishes blood, and coordinates medicines (Nie et al., 2022).

Originally recorded in the classical text *Shi Yi De Xiao Fang* (Efficacious Remedies of the Physicians) as a prescription for treating symptoms such as loss of appetite, vomiting, diarrhea, and indigestion, YGJT has since been widely used for gastrointestinal disorders (Yilin, 2009). Building on this traditional application, it has been investigated for its potential in lung cancer to manage gastrointestinal symptoms caused by the disease itself and by anticancer treatments (Wu et al., 2022). Both preclinical and clinical studies have reported that YGJT increases appetite, protects gastric mucosa, and promotes digestive fluid secretion, thereby addressing appetite loss in individuals with cancer (Sun et al., 2020; Dai et al., 2022; Wu et al., 2022). YGJT prevents anorexia and gastrointestinal dysmotility by antagonizing 5-HT2b/2c receptors in the stomach and hypothalamus, activating the ghrelin receptor GHS-R1a, and inhibiting deacylating enzymes to enhance ghrelin activity (Fujitsuka and Uezono, 2014; Yamada et al., 2021). Additionally, YGJT has demonstrated efficacy in the gastrointestinal system by improving stress-induced gastric hypersensitivity through NO-mediated pathways, promoting gastric accommodation, reducing gastric dysmotility, and inhibiting adrenocorticotropic hormone and cortisol (Inokuchi et al., 2021). In particular, its mechanisms in cancer-induced and chemotherapy-induced anorexia involve activation of ghrelin signaling pathways, antagonism of 5-HT2B/2C receptors, and protection of the gastrointestinal mucosal barrier (Wu et al., 2022; Sun et al., 2024). Through these mechanisms, YGJT has been shown in animal and clinical studies to prevent cancer cachexia and increase food intake following chemotherapy (Fujitsuka and Uezono, 2014; Yamada et al., 2021). This anti-cachectic effect is of high translational value, as cancer-associated cachexia is a major contributor to morbidity and mortality in lung cancer. By improving nutritional status, YGJT may help break the cycle of weight loss and functional decline that limits patients’ tolerance to aggressive anticancer therapies. Previous systematic reviews and meta-analyses have primarily examined the effects of YGJT on upper gastrointestinal symptoms, such as functional dyspepsia and chemotherapy-induced anorexia (Mogami and Hattori, 2014; Hoshino et al., 2019; Ko et al., 2021; Sun et al., 2024; Wang et al., 2024). These findings align with the present study, which demonstrated a reduction in digestive tract reactions, further supporting the effectiveness of YGJT in managing gastrointestinal symptoms in lung cancer treatment.

In the subgroup analysis, JCYGJT exhibited greater efficacy against digestive tract reactions than modified YGJT or HSYGJT. This could be explained by its focus on benefiting Qi and strengthening the spleen through the increased proportion of Atractylodis Macrocephalae Rhizoma as a primary component, combined with the addition of Aurantii Fructus, which regulates Qi and transforms phlegm, and Aucklandiae Radix, which regulates Qi and resolves masses (Bi et al., 2024). These combined effects likely enhanced its digestive properties, enabling better management of gastrointestinal side effects. However, the small number of available studies makes it difficult to draw definitive conclusions about the relative superiority of these formulations. As the synergistic use of herbs is a fundamental principle of traditional East Asian medicine (Wang et al., 2012), further research is needed to better differentiate the effects of YGJT and its modified decoctions and assess their clinical implications.

This study indicates that YGJT may also enhance the effectiveness of antitumor therapy, an area that has been relatively underexplored. This effect is reflected in the observed improvements in ORR and DCR. From a clinical standpoint, the magnitude of this synergy is meaningful. Applying our pooled risk ratio of 1.69 to the baseline control group response rate of 31.1% yields an absolute improvement in ORR of approximately 21.5%. This level of benefit is noteworthy, as more modest improvements in ORR have been considered clinically significant in pivotal lung cancer trials (Cho et al., 2022). There is substantial evidence from preclinical studies that YGJT has antitumor effects. For example, YGJT has been reported to exert antitumor effects in esophageal squamous cell carcinoma by inhibiting the miR-34a/STAT3/IL-6R pathway and enhancing antitumor immune responses by inhibiting PD-1, thereby restoring T-cell cytotoxicity (Han et al., 2023). In liver cancer, YGJT was observed to downregulate UBE2I and the NF-κB/PD-L1 pathway via miR-122-3p (Guo et al., 2024). In NSCLC, YGJT has been suggested to modulate several genes associated with neutrophils in ways that enhance immune responses or mitigate inflammatory side effects, with RNA-induced gene silencing potentially contributing to its anticancer effects (Chen et al., 2017; Xin et al., 2022).

From the perspective of traditional East Asian medicine, the principle of “strengthening the body” and “eliminating evil” suggests that YGJT could potentiate cancer treatment by tonifying Qi, mitigating pathological byproducts such as phlegm dampness, and directly contributing to tumor suppression. Sensitivity analysis using Qi deficiency-related patterns as diagnostic criteria showed improved ORR while reducing the occurrence of adverse events, supporting the clinical relevance of these principles. However, the results for other outcomes were inconsistent, likely because most studies using pattern identification were conducted on advanced lung cancer cases, and the limited number of studies reduced statistical power. Future studies using pattern identification could facilitate robust comparisons between patterns and clarify those most suitable for YGJT in lung cancer treatment.

Furthermore, YGJT resulted in a higher tumor response rate when combined with chemotherapy than when combined with EGFR-TKIs. Although this suggests that YGJT enhances the cytotoxic effects of conventional agents, it is more likely attributable to the more advanced cancer stage in the EGFR-TKI group, possibly related to the indication criteria for EGFR-TKI use. Additional studies examining possible synergistic or antagonistic interactions between YGJT and other Western therapies, such as immunotherapy and radiotherapy, which remain largely unexplored, are warranted. Such research is essential to validate YGJT as a viable adjunct to standard oncological treatments.

A key effect of YGJT in cancer treatment may be its immunomodulatory activity. The role of lymphocyte subsets in cancer prognosis remains incompletely understood, with studies reporting varied results. While CD4^+^ T cells enhance immune responses, CD8^+^ T cells can suppress immune activity and are associated with tumor progression and growth (Wang et al., 2013). CD4^+^ T cells are believed to play a critical role in anti-tumor immunity by directly killing cancer cells, with decreased CD4^+^ levels linked to lower survival rates in patients with lung cancer (Oh and Fong, 2021; Eberst et al., 2022; Malyshkina et al., 2023). The CD4+/CD8+ ratio reflects immune function balance and is an important prognostic biomarker for assessing immune competence in patients with cancer (Xu et al., 2018). Additionally, patients with NSCLC who respond well to anti-PD-1 treatment show increased levels of CD4^+^ T cells and a higher CD4+/CD8+ ratio (Zhuang et al., 2024). Our analysis aligns with these principles, revealing that YGJT significantly increased total T cells (CD3^+^) and CD4^+^ T cells, leading to an elevated CD4+/CD8+ ratio. These findings suggest that YGJT enhances systemic immune function. This enhancement is of high translational value, as it suggests YGJT may counteract the profound T-cell exhaustion and immunosuppressive tumor microenvironment that are key features of lung cancer pathophysiology, potentially restoring the patient’s capacity to respond to both conventional and immune-based therapies.

The decrease in CD8^+^ cell levels observed in this study is controversial, as research on its prognostic significance in lung cancer remains inconclusive. Some studies have indicated that an increase in CD8^+^ T cells is an independent prognostic factor for poor survival in patients with malignant mesothelioma or advanced NSCLC (McCoy et al., 2013), while a systematic review reported that CD3^+^, CD4^+^, and CD8^+^ cells are all positively associated with improved overall survival in patients with NSCLC (Yan et al., 2024). These results suggest that while YGJT may boost immune function and demonstrate anticancer efficacy, the changes in CD8^+^ levels and their effects on prognosis require further investigation through clinical trials.

Tumor markers are closely associated with the disease stage of lung cancer and are widely used for screening, clinical diagnosis, and prognostic assessment (Kumar et al., 2006; Arya and Bhansali, 2011). Specifically, CEA, NSE, SCC, CYFRA 21-1, and CA19-9 have become established indicators for evaluating prognosis in lung cancer, with reductions in these markers often correlating with extended survival (Holdenrieder et al., 2017; Yang et al., 2019; Trulson and Holdenrieder, 2024). In this study, YGJT was shown to reduce these tumor markers. However, due to the high heterogeneity of the included studies, relatively small sample sizes, and lack of data on combination therapy with EGFR-TKIs, further research is required to elucidate the relationship between YGJT and tumor markers.

KPS is one of the most critical prognostic factors affecting the outcomes of patients with cancer, including those with lung cancer (Buccheri et al., 1996; West and Jin, 2015). Therefore, maintaining a high KPS score is crucial in lung cancer treatment. Adding YGJT to conventional therapy resulted in relatively greater improvements in both KPS and symptom scores, suggesting that YGJT not only synergizes with antitumor treatments but also improves patient symptoms, enhances quality of life, and increases treatment tolerability, thereby supporting the overall goals of cancer therapy. The ability of adjunctive YGJT to significantly improve KPS suggests its benefits translate to a tangible enhancement in patients’ functional status and daily life. The mean improvement of 8.62 points observed in our analysis substantially exceeds the 3.83-point threshold empirically identified as the minimal clinically important difference for a noticeable improvement in KPS (Ringash et al., 2007). This indicates that the observed change is not only statistically significant but also represents a highly meaningful clinical benefit for patients.

Another major aspect of the therapeutic effects of YGJT is its reduction of adverse effects associated with cancer treatment. These adverse effects, primarily bone marrow suppression and gastrointestinal toxicity, often lead to a decline in quality of life. YGJT effectively reduced these side effects while improving patients’ quality of life. YGJT’s ability to mitigate a spectrum of adverse events, including both myelosuppression and gastrointestinal toxicity, contrasts with standard supportive care, which often requires the administration of multiple single-target agents. This suggests a potential role for YGJT as a broad-spectrum supportive care intervention, capable of simplifying patient management. *In vivo* studies have reported that YGJT and its modified formulations prevent cisplatin-induced neurotoxicity through antioxidant effects, regulation of mitochondrial function, and alleviation of paclitaxel-induced neuropathy and leukopenia (Chiou et al., 2018; Shiah et al., 2023). Consistent with its immune-balancing properties, YGJT also reduced myelosuppression and leukopenia in this study, suggesting an improvement in immune function that contributes to its anti-tumor effects. Further research on the immunomodulatory effects of YGJT may provide stronger evidence of its potential synergistic effects with current anticancer agents.

The strengths of this study are as follows: To our knowledge, this is the first meta-analysis examining the synergistic effects of YGJT and antitumor therapy in lung cancer patients. This review encompasses a broad range of outcomes, including ORR, DCR, KPS score, clinical symptom score, immune function markers, tumor markers, and treatment-related adverse events, providing a comprehensive assessment of the efficacy and safety of YGJT. This study used a robust data extraction process conducted by multiple independent reviewers to ensure high reliability and minimize bias. Sensitivity analyses specific to advanced-stage lung cancer further reinforced the robustness of the results, while publication bias assessments using funnel plots and Egger’s test added an additional layer of validation. We also conducted an extensive database search without restrictions on language, publication year, or publication type.

Despite its strengths, this review has several limitations. First, a primary limitation of this review is the inability to perform our pre-specified comparative subgroup analyses, particularly for cancer stage and histological subtype. This was due to an insufficient number of included studies representing certain key subgroups (e.g., early-stage or SCLC), which prevented a direct meta-analytic comparison. However, as pre-specified in our protocol, we conducted a sensitivity analysis on the cohort of studies involving advanced-stage cancer, which largely confirmed the robustness of our overall findings for this population. Second, the generalizability of the findings is limited as most studies were conducted in China. This geographical concentration raises questions about the applicability of these results to other ethnic populations. Third, our search strategy, while extensive across published literature databases, did not include a specific search of clinical trial registries or other sources of grey literature. This could have resulted in the omission of relevant unpublished or ongoing trials, representing a potential source of publication bias. Additionally, the majority of the included RCTs were rated as having a “high” or “some concerns” for risk of bias, primarily due to inadequate blinding and unclear randomization methods. This high risk of bias substantially tempers confidence in the observed effect sizes and may have led to an overestimation of the treatment benefit. Furthermore, the substantial clinical heterogeneity, driven by the wide variability in modified YGJT interventions, complicates the attribution of the observed effects to a single, standardized intervention. Future research should aim to address these limitations by including high-quality multicenter RCTs with detailed protocols to validate the findings and explore the application of YGJT in broader, more diverse populations.

## 5 Conclusion

This systematic review provides evidence supporting the efficacy and safety of modified YGJT as an adjuvant therapy for lung cancer. The findings indicate that YGJT improves ORR and DCR, enhances KPS scores and clinical symptoms, boosts immune function, and reduces tumor markers and treatment-related adverse events. Despite these promising results, the overall quality of the included studies was low, with limitations such as small sample sizes, potential biases, and high heterogeneity. These challenges underscore the need for well-designed, large-scale, multicenter RCTs to confirm these findings and further investigate the underlying mechanisms of the effects of YGJT. Future research should aim to address these limitations and explore the interactions between YGJT and contemporary cancer treatments, including immunotherapy and radiotherapy, to fully unlock its potential in comprehensive cancer care.

## Data Availability

The original contributions presented in the study are included in the article/Supplementary Material, further inquiries can be directed to the corresponding author.
