# Supplementary material for: Therapeutic potential of modified Yukgunja-tang (Liujunzi Decoction, Rikkunshito) as an adjuvant treatment for lung cancer: a systematic review and meta-analysis
Source: Front Pharmacol. 2025 Oct 1;16:1657423. doi: 10.3389/fphar.2025.1657423 (PMC12522206; doi:10.3389/fphar.2025.1657423)

Supplementary Material

# Supplementary Tables

Supplementary Table 1. PRISMA 2020 Checklist

| **Section and Topic** | **Item #** | **Checklist item** | **Location where item is reported** |
| --- | --- | --- | --- |
| **TITLE** | | |  |
| Title | 1 | Identify the report as a systematic review. (meta-analysis, or both) | Title |
| **ABSTRACT** | | |  |
| Abstract | 2 | See the PRISMA 2020 for Abstracts checklist. | Abstract |
| **INTRODUCTION** | | |  |
| Rationale | 3 | Describe the rationale for the review in the context of existing knowledge. | Introduction |
| Objectives | 4 | Provide an explicit statement of the objective(s) or question(s) the review addresses. | Introduction |
| **METHODS** | | |  |
|  | | |  |
| Eligibility criteria | 5 | Specify the inclusion and exclusion criteria for the review and how studies were grouped for the syntheses. | Materials and Methods – Eligibility Criteria |
| Information sources | 6 | Specify all databases, registers, websites, organisations, reference lists and other sources searched or consulted to identify studies. Specify the date when each source was last searched or consulted. | Materials and Methods – Search Strategy |
| Search strategy | 7 | Present the full search strategies for all databases, registers and websites, including any filters and limits used. | Materials and Methods – Search Strategy |
| Selection process | 8 | Specify the methods used to decide whether a study met the inclusion criteria of the review, including how many reviewers screened each record and each report retrieved, whether they worked independently, and if applicable, details of automation tools used in the process. | Materials and Methods – Study Selection and Data Extraction |
| Data collection process | 9 | Specify the methods used to collect data from reports, including how many reviewers collected data from each report, whether they worked independently, any processes for obtaining or confirming data from study investigators, and if applicable, details of automation tools used in the process. | Materials and Methods – Study Selection and Data Extraction |
| Data items | 10a | List and define all outcomes for which data were sought. Specify whether all results that were compatible with each outcome domain in each study were sought (e.g. for all measures, time points, analyses), and if not, the methods used to decide which results to collect. | Materials and Methods – Eligibility Criteria |
|  | 10b | List and define all other variables for which data were sought (e.g. participant and intervention characteristics, funding sources). Describe any assumptions made about any missing or unclear information. | Materials and Methods – Study Selection and Data Extraction |
| Study risk of bias assessment | 11 | Specify the methods used to assess risk of bias in the included studies, including details of the tool(s) used, how many reviewers assessed each study and whether they worked independently, and if applicable, details of automation tools used in the process. | Materials and Methods – Quality Assessment |
| Effect measures | 12 | Specify for each outcome the effect measure(s) (e.g. risk ratio, mean difference) used in the synthesis or presentation of results. | Materials and Methods – Statistical Analysis |
| Synthesis methods | 13a | Describe the processes used to decide which studies were eligible for each synthesis (e.g. tabulating the study intervention characteristics and comparing against the planned groups for each synthesis (item #5)). | Materials and Methods – Statistical Analysis |
|  | 13b | Describe any methods required to prepare the data for presentation or synthesis, such as handling of missing summary statistics, or data conversions. | Materials and Methods – Statistical Analysis |
|  | 13c | Describe any methods used to tabulate or visually display results of individual studies and syntheses. | Materials and Methods – Statistical Analysis |
|  | 13d | Describe any methods used to synthesize results and provide a rationale for the choice(s). If meta-analysis was performed, describe the model(s), method(s) to identify the presence and extent of statistical heterogeneity, and software package(s) used. | Materials and Methods – Statistical Analysis |
|  | 13e | Describe any methods used to explore possible causes of heterogeneity among study results (e.g. subgroup analysis, meta-regression). | Materials and Methods – Subgroup Analysis and Assessment of Heterogeneity |
|  | 13f | Describe any sensitivity analyses conducted to assess robustness of the synthesized results. | Materials and Methods – Sensitivity Analysis |
| Reporting bias assessment | 14 | Describe any methods used to assess risk of bias due to missing results in a synthesis (arising from reporting biases). | Materials and Methods – Statistical Analysis |
| Certainty assessment | 15 | Describe any methods used to assess certainty (or confidence) in the body of evidence for an outcome. | Materials and Methods –Certainty of Evidence |
| **RESULTS** | | |  |
| Study selection | 16a | Describe the results of the search and selection process, from the number of records identified in the search to the number of studies included in the review, ideally using a flow diagram. | Results – Retrieval Results |
|  | 16b | Cite studies that might appear to meet the inclusion criteria, but which were excluded, and explain why they were excluded. | Results – Retrieval Results |
| Study characteristics | 17 | Cite each included study and present its characteristics. | Results - Characteristics of Included Studies |
| Risk of bias in studies | 18 | Present assessments of risk of bias for each included study. | Results – Risk of Bias |
| Results of individual studies | 19 | For all outcomes, present, for each study: (a) summary statistics for each group (where appropriate) and (b) an effect estimates and its precision (e.g. confidence/credible interval), ideally using structured tables or plots. | Results |
| Results of syntheses | 20a | For each synthesis, briefly summarise the characteristics and risk of bias among contributing studies. | Results |
|  | 20b | Present results of all statistical syntheses conducted. If meta-analysis was done, present for each the summary estimate and its precision (e.g. confidence/credible interval) and measures of statistical heterogeneity. If comparing groups, describe the direction of the effect. | Results |
|  | 20c | Present results of all investigations of possible causes of heterogeneity among study results. | Results |
|  | 20d | Present results of all sensitivity analyses conducted to assess the robustness of the synthesized results. | Results – Sensitivity Analysis |
| Reporting biases | 21 | Present assessments of risk of bias due to missing results (arising from reporting biases) for each synthesis assessed. | Results |
| Certainty of evidence | 22 | Present assessments of certainty (or confidence) in the body of evidence for each outcome assessed. | Results – Certainty of Evidence |
| **DISCUSSION** | | |  |
| Discussion | 23a | Provide a general interpretation of the results in the context of other evidence. | Discussion |
|  | 23b | Discuss any limitations of the evidence included in the review. | Discussion |
|  | 23c | Discuss any limitations of the review processes used. | Discussion |
|  | 23d | Discuss implications of the results for practice, policy, and future research. | Discussion |
| **OTHER INFORMATION** | | |  |
| Registration and protocol | 24a | Provide registration information for the review, including register name and registration number, or state that the review was not registered. | Materials and Methods |
|  | 24b | Indicate where the review protocol can be accessed, or state that a protocol was not prepared. | Materials and Methods |
|  | 24c | Describe and explain any amendments to information provided at registration or in the protocol. | Materials and Methods |
| Support | 25 | Describe sources of financial or non-financial support for the review, and the role of the funders or sponsors in the review. | Funding |
| Competing interests | 26 | Declare any competing interests of review authors. | Conflict of Interest |
| Availability of data, code and other materials | 27 | Report which of the following are publicly available and where they can be found: template data collection forms; data extracted from included studies; data used for all analyses; analytic code; any other materials used in the review. | Data Availability Statement |

Supplementary Table 2. Search strategy used in each database

**Medline via PubMed**

|  | Searches | Results |
| --- | --- | --- |
| #1 | "Lung Neoplasms"[Mesh] | 294016 |
| #2 | Pulmonary Neoplasm*[TIAB] OR Lung Neoplasm*[TIAB] OR Lung Cancer*[TIAB] OR Cancer of Lung[TIAB] OR Pulmonary Cancer*[TIAB] OR Cancer of the Lung[TIAB] OR Lung Carcinoma*[TIAB] OR Ca Lung[TIAB] OR Carcinogenesis of the Lung[TIAB] OR Lung Malignancies[TIAB] OR Lung Malignancy[TIAB] OR Malignancies of the Lung[TIAB] OR Malignancy of the Lung[TIAB] OR Malignant Neoplasm of the Lung[TIAB] OR Malignant Tumor of the Lung[TIAB] OR Pulmonary Malignancies[TIAB] OR Pulmonary Malignancy[TIAB] OR Schneeberg Disease[TIAB] OR Schneeberg Lung Disease[TIAB] OR Lung Tumo*[TIAB] OR Pulmonary Carcinoma*[TIAB] OR Carcinoma of Lung[TIAB] OR Carcinoma of the Lung[TIAB] OR Carcinoma Pulmomum[TIAB] OR Carcinomatosis of the Lung[TIAB] OR Carcinomatous Lung[TIAB] OR Carcinomatous Pulmonary[TIAB] OR Non Small Cell Lung Carcinoma*[TIAB] OR Non-Small Cell Lung Cancer*[TIAB] OR Non-Small-Cell Lung Carcinoma*[TIAB] OR Non-Small-Cell Lung Cancer*[TIAB] OR Non-Small Cell Lung Carcinoma*[TIAB] OR Non-Small Cell Lung Cancer*[TIAB] OR Nonsmall Cell Lung Cancer*[TIAB] OR Nonsmall Cell Lung Carcinoma*[TIAB] OR NSCLC[TIAB] OR Small Cell Carcinoma*[TIAB] OR Oat Cell Carcinoma*[TIAB] OR Oat Cell Lung Carcinoma*[TIAB] OR Oat Cell Lung Cancer*[TIAB] OR Oat Cell Cancer*[TIAB] OR SCLC[TIAB] OR Small Cell Lung Cancer*[TIAB] OR Small Cell Lung Carcinoma*[TIAB] | 278277 |
| #3 | #1 OR #2 | 388555 |
| #4 | Yukgunja[TIAB] OR Yukgunja-tang[TIAB] OR Liujunzi[TIAB] OR “Liu jun zi”[TIAB] OR Liu-jun-zi[TIAB] OR Liu-jun-zi-jia-jian[TIAB] OR Liujunzi-tang[TIAB] OR Liu-junzi-tang[TIAB] OR Liu-jun-zi-tang[TIAB] OR LZJT[TIAB] OR LZJD[TIAB] OR Xiangshaliujunzi[TIAB] OR Xiangshaliujunzi-tang[TIAB] OR “Xiangsha liujunzi”[TIAB] OR “Xiang sha liu jun zi”[TIAB] OR Xiang-sha-liu-jun-zi[TIAB] OR Xiang-sha-liu-jun-zi-tang[TIAB] OR Xiang-sha-liu-jun-zi-jia-jian[TIAB] OR “Rik kun shi”[TIAB] OR Rik-kun-shi-to[TIAB] OR Rikkun-shi-to[TIAB] OR Rikkunshi-to[TIAB] OR Rikkunshi[TIAB] OR Rikkunshito[TIAB] OR TJ-43[TIAB] OR Yukgunja*[TIAB] OR Liujunzi*[TIAB] OR Rikkunshito*[TIAB] OR Rikkunshi‐to*[TIAB] | 313 |
| #5 | (groups[TIAB] OR trial[TIAB] OR randomly[TIAB] OR "drug therapy"[SH] OR placebo[TIAB] OR randomized[TIAB] OR "controlled clinical trial"[PT] OR "randomized controlled trial"[PT]) NOT (animals[MH] NOT (humans[MH] AND animals[MH])) | 5539022 |
| #6 | #3 AND #4 AND #5 | **9** |

**EMBASE**

|  | Searches | Results |
| --- | --- | --- |
| #1 | 'lung tumor'/exp | 612720 |
| #2 | 'pulmonary neoplasm*':ab,ti OR 'lung neoplasm*':ab,ti OR 'lung cancer*':ab,ti OR 'cancer of lung':ab,ti OR 'pulmonary cancer*':ab,ti OR 'cancer of the lung':ab,ti OR 'lung carcinoma*':ab,ti OR 'ca lung':ab,ti OR 'carcinogenesis of the lung':ab,ti OR 'lung malignancies':ab,ti OR 'lung malignancy':ab,ti OR 'malignancies of the lung':ab,ti OR 'malignancy of the lung':ab,ti OR 'malignant neoplasm of the lung':ab,ti OR 'malignant tumor of the lung':ab,ti OR 'pulmonary malignancies':ab,ti OR 'pulmonary malignancy':ab,ti OR 'schneeberg disease':ab,ti OR 'schneeberg lung disease':ab,ti OR 'lung tumo*':ab,ti OR 'pulmonary carcinoma*':ab,ti OR 'carcinoma of lung':ab,ti OR 'carcinoma of the lung':ab,ti OR 'carcinoma pulmomum':ab,ti OR 'carcinomatosis of the lung':ab,ti OR 'carcinomatous lung':ab,ti OR 'carcinomatous pulmonary':ab,ti OR 'non small cell lung carcinoma*':ab,ti OR 'non small cell lung cancer*':ab,ti OR 'non-small-cell lung carcinoma*':ab,ti OR 'non-small-cell lung cancer*':ab,ti OR 'non-small cell lung carcinoma*':ab,ti OR 'non-small cell lung cancer*':ab,ti OR 'nonsmall cell lung cancer*':ab,ti OR 'nonsmall cell lung carcinoma*':ab,ti OR 'nsclc':ab,ti OR 'small cell carcinoma*':ab,ti OR 'oat cell carcinoma*':ab,ti OR 'oat cell lung carcinoma*':ab,ti OR 'oat cell lung cancer*':ab,ti OR 'oat cell cancer*':ab,ti OR 'sclc':ab,ti OR 'small cell lung cancer*':ab,ti OR 'small cell lung carcinoma*':ab,ti | 413665 |
| #3 | #1 OR #2 | 659871 |
| #4 | yukgunja:ab,ti OR 'yukgunja tang':ab,ti OR liujunzi:ab,ti OR ‘liu jun zi’:ab,ti OR 'liu jun zi jia jian':ab,ti OR 'liujunzi tang':ab,ti OR 'liu junzi tang':ab,ti OR 'liu jun zi tang':ab,ti OR lzjt:ab,ti OR lzjd:ab,ti OR xiangshaliujunzi:ab,ti OR 'xiangshaliujunzi tang':ab,ti OR 'xiangsha liujunzi':ab,ti OR 'xiang sha liu jun zi':ab,ti OR 'xiang sha liu jun zi tang':ab,ti OR 'xiang sha liu jun zi jia jian':ab,ti OR 'rik kun shi':ab,ti OR 'rik kun shi to':ab,ti OR 'rikkun shi to':ab,ti OR 'rikkunshi to':ab,ti OR rikkunshi:ab,ti OR rikkunshito:ab,ti OR 'tj-43':ab,ti OR yukgunja*:ab,ti OR liujunzi*:ab,ti OR rikkunshito*:ab,ti OR rikkunshi‐to*:ab,ti | 487 |
| #5 | 'crossover procedure'/exp OR 'double blind procedure'/exp OR 'randomized controlled trial'/exp OR 'single blind procedure'/exp OR random* OR factorial* OR crossover* OR 'cross over' OR 'cross-over' OR placebo* OR (doubl* AND blind*) OR (singl* AND blind*) OR assign* OR allocat* OR volunteer* | 3538410 |
| #6 | #3 AND #4 AND #5 | **12** |

**Cochrane Library**

|  | Searches | Results |
| --- | --- | --- |
| #1 | MeSH descriptor: [Neoplasms] explode all trees | 126239 |
| #2 | (Pulmonary NEXT Neoplasm* OR Lung NEXT Neoplasm* OR Lung NEXT Cancer* OR "Cancer of Lung" OR Pulmonary NEXT Cancer* OR "Cancer of the Lung" OR Lung NEXT Carcinoma* OR "Ca Lung" OR Carcinogenesis NEXT of NEXT the NEXT Lung OR Lung NEXT Malignancies OR Lung NEXT Malignancy OR Malignancies NEXT of NEXT the NEXT Lung OR Malignancy NEXT of NEXT the NEXT Lung OR Malignant NEXT Neoplasm NEXT of NEXT the NEXT Lung OR Malignant NEXT Tumor NEXT of NEXT the NEXT Lung OR Pulmonary NEXT Malignancies OR Pulmonary NEXT Malignancy OR Schneeberg NEXT Disease OR Schneeberg NEXT Lung NEXT Disease OR Lung NEXT Tumo* OR Pulmonary NEXT Carcinoma* OR Carcinoma NEXT of NEXT Lung OR Carcinoma NEXT of NEXT the NEXT Lung OR Carcinoma NEXT Pulmomum OR Carcinomatosis NEXT of NEXT the NEXT Lung OR Carcinomatous NEXT Lung OR Carcinomatous NEXT Pulmonary OR Non NEXT Small NEXT Cell NEXT Lung NEXT Carcinoma* OR Non NEXT Small NEXT Cell NEXT Lung NEXT Cancer* OR Non-Small-Cell NEXT Lung NEXT Carcinoma* OR Non-Small-Cell NEXT Lung NEXT Cancer* OR Non NEXT Small NEXT Cell NEXT Lung NEXT Carcinoma* OR Non NEXT Small NEXT Cell NEXT Lung NEXT Cancer* OR Nonsmall NEXT Cell NEXT Lung NEXT Cancer* OR Nonsmall NEXT Cell NEXT Lung NEXT Carcinoma* OR NSCLC OR Small NEXT Cell NEXT Carcinoma* OR Oat NEXT Cell NEXT Carcinoma* OR Oat NEXT Cell NEXT Lung NEXT Carcinoma* OR Oat NEXT Cell NEXT Lung NEXT Cancer* OR Oat NEXT Cell NEXT Cancer* OR SCLC OR Small NEXT Cell NEXT Lung NEXT Cancer* OR Small NEXT Cell NEXT Lung NEXT Carcinoma*):ti,ab,kw | 29783 |
| #3 | #1 OR #2 | 143073 |
| #4 | (Yukgunja OR Yukgunja-tang OR Liujunzi OR ‘Liu jun zi’ OR Liu-jun-zi OR Liu-jun-zi-jia-jian OR Liujunzi-tang OR Liu-junzi-tang OR Liu-jun-zi-tang OR LZJT OR LZJD OR Xiangshaliujunzi OR Xiangshaliujunzi-tang OR ‘Xiangsha liujunzi’ OR ‘Xiang sha liu jun zi’ OR Xiang-sha-liu-jun-zi OR Xiang-sha-liu-jun-zi-tang OR Xiang-sha-liu-jun-zi-jia-jian OR ‘Rik kun shi’ OR Rik-kun-shi-to OR Rikkun-shi-to OR Rikkunshi-to OR Rikkunshi OR Rikkunshito OR TJ-43 OR Yukgunja* OR Liujunzi* OR Rikkunshito* OR Rikkunshi‐to*):ti,ab,kw | 189 |
| #5 | #3 AND #4 | **23** |

**Oriental Medicine Advanced Searching Integrated System**

|  | Searches | Results |
| --- | --- | --- |
| #1 | (폐종양\|폐암) AND (육군자\|육군자탕\|향사육군자\|향사육군자탕\|육군자가감) | **0** |

**Korean Studies Information Service System**

|  | Searches | Results |
| --- | --- | --- |
| #1 | 전체 = (폐종양\|폐암) AND 전체 = (육군자\|육군자탕\|향사육군자\|향사육군자탕\|육군자가감) | **0** |

**Research Information Sharing Service**

|  | Searches | Results |
| --- | --- | --- |
| #1 | 전체 : 폐종양\|폐암 <AND> 전체 : 육군자\|육군자탕\|향사육군자\|향사육군자탕\|육군자가감 | **1** |

**ScienceON**

|  | Searches | Results |
| --- | --- | --- |
| #1 | 전체=폐종양\|폐암 AND 전체=육군자\|육군자탕\|향사육군자\|향사육군자탕\|육군자가감 | **0** |

**KMBASE**

|  | Searches | Results |
| --- | --- | --- |
| #1 | (폐종양\|abstract) OR (폐암\|abstract) | 875 |
| #2 | (육군자\|abstract) OR (육군자탕\|abstract) OR (향사육군자\|abstract) OR (향사육군자탕\|abstract) OR (육군자가감\|abstract) | 10 |
| #3 | #1 AND #2 | **0** |

**China National Knowledge Infrastructure Database**

|  | Searches | Results |
| --- | --- | --- |
| #1 | (SU='肺肿瘤' OR TKA='肺肿瘤' OR SU='肺癌' OR TKA='肺癌') AND (SU='六君子' OR TKA='六君子' OR SU='六君子汤' OR TKA='六君子汤' SU='香砂六君子' OR TKA='香砂六君子' OR SU='香砂六君子汤' OR TKA='香砂六君子汤' OR SU='六君子加減' OR TKA='六君子加減') AND (SU='随机' OR TKA='随机' OR SU='对照' OR TKA='对照' OR SU='Randomized' OR TKA='Randomized') | **101** |

**CiNii**

|  | Searches | Results |
| --- | --- | --- |
| #1 | (肺腫瘍 OR 肺癌) AND (六君子 OR 六君子湯 OR 香砂六君子 OR 香砂六君子湯 OR 六君子加減) | **1** |

Supplementary Table 3. Excluded studies and reasons after full-text review

Study design does not meet review criteria (n = 4)

| **Author(Year)** | **Title** |
| --- | --- |
| Oteki (2016) | Effect of rikkunshi-to treatment on chemotherapy-induced appetite loss in patients with lung cancer: A prospective study |
| Su (2018) | Clinical Observation of Modified Liu Jun Zi Tang as an Adjuvant to Chemotherapy in the Treatment of Advanced Non-Small Cell Lung Cancer |
| Cai (2019) | Clinical effect of Zhijie Liujunzi decoction in the treatment of cancer-related fatigue of advanced lung cancer |
| Yoshiya (2020) | Prospective, randomized, cross-over pilot study of the effects of Rikkunshito, a Japanese traditional herbal medicine, on anorexia and plasma-acylated ghrelin levels in lung cancer patients undergoing cisplatin-based chemotherapy |

Not a research paper (n = 2)

| **Author(Year)** | **Title** |
| --- | --- |
| Yoshiya (2016) | The effect of rikkunshito, a traditional Japanese herbal medicine, on food intake and plasma acylated ghrelin levels in lung cancer patients treated with platinum-based chemotherapy |
| Suzuki (2019) | Metabolites as mediator for alleviating adverse effects in chemotherapy: A new finding from a recent study of Japanese traditional Kampo medicine, rikkunshito (RKT) |

Intervention does not meet review criteria (n = 2)

| **Author(Year)** | **Title** |
| --- | --- |
| Han (2019) | EFFECT OF SIX JUNZI DECOCTION ON ADVANCED NSCLC |
| Shao (2019) | The Clinical Efficacy of Zhijie Liu Junzi Decoction in Treating Cancer-Related Fatigue in Patients with Advanced Stage Lung Cancer |

Supplementary Table 4. List of components of modified YGJT in Chinese characters

| Name | Author (Year) | Main components | Modified part |
| --- | --- | --- | --- |
| Modified YGJT | Fang et al. (2015) | 炒黨參 30 g, 炒白朮 10 g, 茯苓 15 g, 甘草 3 g, 制半夏 9 g, 陳皮 10 g, 大棗 30 g | 雞血藤 30 g, 仙茅 15 g, 淫羊藿 15 g, 女貞子 30 g, 黃耆 30 g, 當歸 15 g |
| Modified YGJT | Ye (2015) | 人參 9 g, 白朮 12 g, 茯苓 12 g, 甘草 6 g, 陳皮 6 g, 薑半夏 6 g, 生薑 3片 | 山藥 15 g, 薑竹茹 12 g, 炒神曲 12 g, 炒山楂 15 g 1) Diarrhea: add 沙參 6 g, 馬齒莧 15 g 2) Constipation: add 瓜蔞 15 g, 麻子仁 6 g 3) Oral inflammation: add 生地 12 g, 黃連 3 g |
| Modified YGJT | Zhou et al. (2015) | 黨參 30 g, 茯苓 15 g, 白朮 15 g, 薑半夏 10 g, 陳皮 10 g, 甘草 5 g, 大棗 10 g, 生薑 3片 | During chemotherapy 1) Cough with sputum: add 浙貝母 15 g, 苦杏仁 10 g, 百部 10 g 2) Constipation: add 酒大黃 10 g, 枳殼 10 g 3) Nausea and vomiting: add 旋覆花 10 g, 赭石 30 g 4) Significant fatigue: add 生曬參 10 g, 黃耆 30 g 5) Poor appetite: add 炒穀芽 30 g, 炒麥芽 30 g, 雞內金 10 g 6) Leukopenia: add 紫河車 10 g, 女貞子 15 g 7) Loose stools: add 藿香 10 g, 砂仁 3 g During the chemotherapy interval 1) Appropriately select medicines for transforming phlegm, resolving masses, and eliminating stasis: add 龍葵 20 g, 天龍 4條, 浙貝母 10 g, 夏枯草 10 g, 莪術 10 g, 鱉甲 10 g, 貓爪草 15 g |
| HSYGJT | Li et al. (2016) | 黨參 15 g, 白朮 15 g, 茯苓 15 g, 半夏 10 g, 陳皮 10 g, 廣木香 10 g, 砂仁 10 g, 甘草 5 g | - |
| Modified YGJT | Xing and Zhai (2016) | 黨參 15 g, 白朮 10 g, 茯苓 10 g, 半夏 10 g, 陳皮 10 g, 甘草 3 g | 黃耆 15 g, 桔梗 10 g, 薏苡仁 15 g, 川貝 5 g, 杏仁 10 g |
| Modified YGJT | Xiong and Xiong (2016) | 黨參 30 g, 茯苓 15 g, 白朮 15 g, 薑半夏 10 g, 陳皮 10 g, 甘草 5 g | 旋覆花 30 g, 浙貝母 15 g, 百部 10 g, 枳殼 10 g, 紫河車 10 g, 苦杏仁 10 g |
| YGJT | Chen et al. (2017) | 人參 5 g, 白朮 5 g, 茯苓 5 g, 半夏 5 g, 甘草 2.5 g, 陳皮 2.5 g, 生薑 2.5 g, 大棗 2.5 g, 澱粉 7.8 g per 100.00 g powder with a crude drug extract ratio of 30.00:7.80 | - |
| Modified YGJT | Fu and Jiang (2017) | 黨參 15 g, 白朮 15 g, 茯苓 15 g, 甘草 10 g, 陳皮 20 g, 清半夏 12 g | 1) Significant pain: add 延胡索, 天仙藤 2) Dysphagia: add 旋覆花, 代赭石 3) Vomiting of acid, phlegm and saliva: add 黃連, 吳茱萸 4) Insomnia: add 酸棗仁, 夜交藤 |
| Modified YGJT | Li et al. (2017) | 黨參 15 g, 茯苓 15 g, 白朮 10 g, 法半夏 10 g, 橘絡 10 g, 甘草 6 g | 黃耆 15 g, 黃精 15 g, 五味子 10 g, 桔梗 10 g, 半枝蓮 30 g, 白花蛇舌草 30 g 1) Severe shortness of breath and coughing due to qi deficiency: add 西洋參 10 g 2) Phlegm, toxin, and stasis accumulation: add 露蜂房 10 g, 白僵蠶 10 g, 桃仁 10 g 3) Nausea and vomiting: add 旋覆花 15 g, 赭石 15 g 4) Leukopenia: add 紫河車 10 g, 女貞子 10 g |
| Modified YGJT | Liu et al. (2017) | 黨參 30 g, 茯苓 15 g, 白朮 15 g, 薑半夏 10 g, 陳皮 10 g, 甘草 5 g | 旋覆花 10 g, 浙貝母 15 g, 百部 10 g, 枳殼 10 g, 紫河車 10 g, 苦杏仁 10 g |
| Modified YGJT | Yang et al. (2017) | 人參 15 g, 茯苓 30 g, 白朮 20 g, 陳皮 12 g, 半夏 12 g, 甘草 6 g | 黃耆 40 g, 薏苡仁 50 g, 菌靈芝 50 g, 女貞子 30 g, 枸杞子 30 g, 鬱金 30 g, 建曲 20 g, 麥芽 30 g |
| Modified YGJT | Zhao et al. (2017) | 薑半夏 15 g, 陳皮 10 g, 黨參 15 g, 茯苓 15 g, 炒白朮 15 g, 甘草 6 g | 白花蛇舌草 30 g, 半枝蓮 15 g, 白英 15 g 1) Severe cough: add 枇杷葉 15 g, 百部 15 g 2) Loss of appetite: add 神曲 15 g, 穀芽 15 g 3) Chest tightness and pain: add 全瓜蔞 30 g, 薤白 10 g, 旋覆花 10 g 4) Blood in phlegm: add 白及 10 g, 白茅根 15 g, 仙鶴草 15 g 5) Pleural effusion: add 葶藶子 15 g, 龍葵 30 g |
| Modified YGJT | Dong et al. (2018) | 薑半夏 15 g, 炒白朮 15 g, 茯苓 15 g, 黨參 15 g, 陳皮 10 g, 甘草 6 g | 白英 15 g, 半枝蓮 15 g, 白花蛇舌草 30 g |
| Modified YGJT | Duan (2018) | 茯苓 9 g, 白朮 9 g, 人參 9 g, 甘草 10 g, 半夏 4.5 g, 陳皮 3 g | 1) Severe cough: add 枇杷葉 15 g, 百部 15 g 2) Chest pain or chest discomfort: add 旋覆花 10 g, 全瓜蔞 30 g, 薤白 10 g  3) Blood in sputum: add 仙鶴草 15 g, 白芨 10 g, 白茅根 15 g |
| YGJT | Harada et al. (2018a, b) | 蒼朮, 人參, 半夏, 茯苓, 大棗, 陳皮, 甘草, 生薑 | - |
| Modified YGJT | Li (2018) | 茯苓 15 g, 黨參 15 g, 法半夏 15 g, 陳皮 10 g, 蒼朮 10 g, 甘草 5 g | 全瓜蔞 10 g, 薏苡仁 30 g 1) Vexation, phlegm heat, and poor sleep: add 炒枳實, 竹茹 2) Phlegm obstruction with shortness of breath: add 炒枳實, 膽南星 3) Thick yellow phlegm: add 黃連, 瓜蔞 4) Chest and flank distention and pain: add 鬱金, 枳實 |
| Modified YGJT | Liu et al. (2018) | 人參 12 g, 白朮 12 g, 茯苓 12 g, (灸) 甘草 6 g, 陳皮 6 g, 半夏 6 g | 1) Severe cough: add 杏仁 6 g, 桔梗 6 g 2) Haemoptysis: add 仙鶴草 6 g, 白茅根 6 g 3) Severe chest pain: add 元胡 6 g, 白芍 6 g |
| Modified YGJT | Sun (2020) | 人參 12 g, 白朮 14 g, 茯苓 14 g, 甘草 6 g, 陳皮 6 g, 半夏 6 g | 1) Cough: add 浙貝母 10 g, 苦杏仁 10 g 2) Nausea and vomiting: add 旋覆花 10 g, 質赭石 15 g 3) Fatigue: add 生曬參 15 g, 黃耆 15 g 4) Leukopenia: add 女貞子 10 g, 紫河車 15 g 5) Loose stools: add 砂仁 5 g, 藿香 10 g |
| Modified YGJT | Sun et al. (2021) | 甘草 6 g, 陳皮 6 g, 半夏 6 g, 人參 12 g, 白朮 14 g, 茯苓 14 g | 1) Cough: add 浙貝母 10 g, 苦杏仁 10 g 2) Nausea and vomiting: add 旋覆花 10 g, 質赭石 15 g 3) Fatigue: add 生曬參 15 g, 黃耆 15 g 4) Leukopenia: add 女貞子 10 g, 紫河車 15 g 5) Loose stools: add 砂仁 5 g, 藿香 10 g |
| Modified YGJT | Zhao et al. (2021) | 黨參 30 g, 白朮 15 g, 茯苓 20 g, 甘草 10 g, 陳皮 5 g, 法半夏 15 g | 1) Severe phlegm-heat: add 膽南星 15 g, 全瓜蔞 15 g 2) Excessive dampness: add 薏苡仁 30 g, 豬苓 20 g 3) Phlegm and stasis accumulation: add 守宮 6 g, 地龍 3 g |
| JCYGJT | Cai et al. (2022) | 人參 10 g, 白朮 20 g, 茯苓 20 g, 半夏 15 g, 陳皮 15 g, 枳殼 20 g, 木香 15 g, 生薑 10 g, 大棗 五枚, 甘草 6 g | - |
| Modified YGJT | Chen et al. (2022) | 人參 10 g, 茯苓 15 g, 法半夏 10 g, 炒白朮 15 g, 陳皮 10 g, 甘草 3 g | 黃耆 20 g, 白花蛇舌草 30 g, 生牡蠣 15 g, 山慈菇 30 g |
| Modified YGJT | Gao (2022) | 人參 12 g, 半夏 6 g, 白朮 12 g, 陳皮 6 g, 茯苓 12 g, 甘草 6 g | 1) Severe cough: add 苦杏仁 6 g, 桔梗 6 g 2) Hemoptysis: add 仙鶴草 6 g, 白茅根 6 g 3) Severe chest pain: add 延胡索 6 g, 白芍 6 g |
| Modified HSYGJT | Guo et al. (2022) | 人參 3 g, 白朮 6 g, 茯苓 6 g, 甘草 2 g, 陳皮 2.5 g, 半夏 3 g, 砂仁 2.5 g, 木香 2 g | 1) Severe abdominal pain: add 延胡索 10 g 2) Severe cold-dampness: add 肉桂 10 g 3) Acid reflux: add 海螵蛸 10 g |
| Modified YGJT | Nie et al. (2022) | 茯苓 15 g, 白朮 15 g, 人參 15 g, 半夏 10 g, 陳皮 10 g, 甘草 6 g | 1) Chest pain: add 白芍 20 g, 枳實 10 g, 桃仁 10 g 2) Haemoptysis: add 白茅根 20 g, 仙鵝草 15 g 3) Yellow phlegm and cough: add 桔梗 10 g, 黃芩 10 g, 梔子 15 g |
| Modified JGYGJT | Wang et al. (2023) | 炒麥芽 30 g, 黨參 20 g, 茯苓 20 g, 烏賊骨 15 g, 浙貝母 15 g, 枳實 10 g, 白朮 10 g, 桔梗 10 g, 半夏 10 g, 陳皮 10 g, 甘草 6 g | 黃耆 30 g, 雞內金 15 g, 預知子 10 g, 防風 6 g |
| Modified HSYGJT | Liu et al. (2023) | 黨參 30 g, 白朮 15 g, 茯苓 10 g, 清半夏 10 g, 陳皮 10 g, 木香 10 g, 砂仁 10 g, 甘草 6 g | 生黃耆 30 g, 桔梗 10 g, 杏仁 10 g 1) Cough and sputum: add 桑白皮, 川貝, 生薏仁, 萊菔子  2) Shortness of breath and chest tightness: add 瓜蔞, 枳實, 薤白  3) Short breath and fatigue: add 蛤蚧, 五味子, 枸杞  4) Nausea and vomiting: add 枳實, 代赭石  5) Epigastric discomfort and acid reflux: add 白及, 烏賊骨  6) Cold stomach and bitter taste: add 乾薑, 黃連  7) Loose stools and bitter taste: add 吳茱萸, 黃連  8) Dry stools: add 柏子仁, 火麻仁, 紫菀  9) Dark tongue and choppy pulse: add 赤芍, 莪術  10) Granulocytopenia: add 女貞子, 旱蓮草, 仙靈脾, 鹿角膠  11) Severe anemia: add 阿膠, 當歸, 雞血藤, 大棗  12) Significant thrombocytopenia: add 山萸肉, 仙鶴草 |
| Modified YGJT | Tao and Liu (2023) | 人參, 白朮, 茯苓, 甘草, 陳皮, 半夏 | 黃耆 20 g, 人參 15 g, 茯苓 15 g, 白芍 15 g, 枸杞 15 g, 黃精 15 g, 菟絲子 15 g, 沙參 15 g, 陳皮 15 g, 白朮 10 g, 半夏 10 g, 甘草 10 g, 麥冬 10 g |
| Modified YGJT | Wang et al. (2023) | 甘草 12 g, 半夏 12 g, 陳皮 15 g, 白朮 15 g, 黨參 15 g, 茯苓 20 g, 大棗 6 枚, 生薑 10 g | 砂仁 6 g, 木香 10 g |
| JCYGJT | Bi et al. (2024) | 人參 9 g, 白朮 10 g, 茯苓 10 g, 半夏 10 g, 陳皮 10 g, 枳殼 20 g, 木香 15 g, 生薑 10 g, 大棗 3 枚, 炙甘草 6 g |  |
| Modified YGJT | Lin et al. (2024) | 白朮 12 g, 茯苓 15 g, 黨參 15 g, 陳皮 9 g, 法半夏 12 g | 石上柏 30 g, 白花蛇舌草 30 g, 石見穿 30 g, 八月札 15 g, 夏枯草 15 g, 天南星 15 g, 干蟾皮 9 g 1) Severe cough: add 枇杷葉 15 g, 百部 15 g 2) Loss of appetite: add 神曲 15 g, 穀芽 15 g 3) Nausea and vomiting: add 旋覆花 15 g, 代赭石 15 g 4) Chest tightness and pain: add 全瓜蔞 30 g, 薤白 10 g, 旋覆花 10 g 5) Pleural effusion: add 葶藶子 15 g, 龍葵 30 g 6) Leukopenia: add 紫河車 10 g, 女貞子 10 g |

HSYGJT, Hyangsayukgunja-tang; JCYGJT, Jichulyukgunja-tang; JGYGJT, Jigilyukgunja-tang; YGJT, Yukgunja-tang.

# Supplementary Figure

Supplementary Figure 1. Funnel plots assessing publication bias. (A) CD4+. (B) CD8+. (C) CD4+/CD8+. (D) CEA. (E) Digestive tract reactions. HSYGJT, Hyangsayukgunja-tang; JCYGJT, Jichulyukgunja-tang; MD, mean difference; RR, risk ratio; SE, standard error; YGJT, Yukgunja-tang.


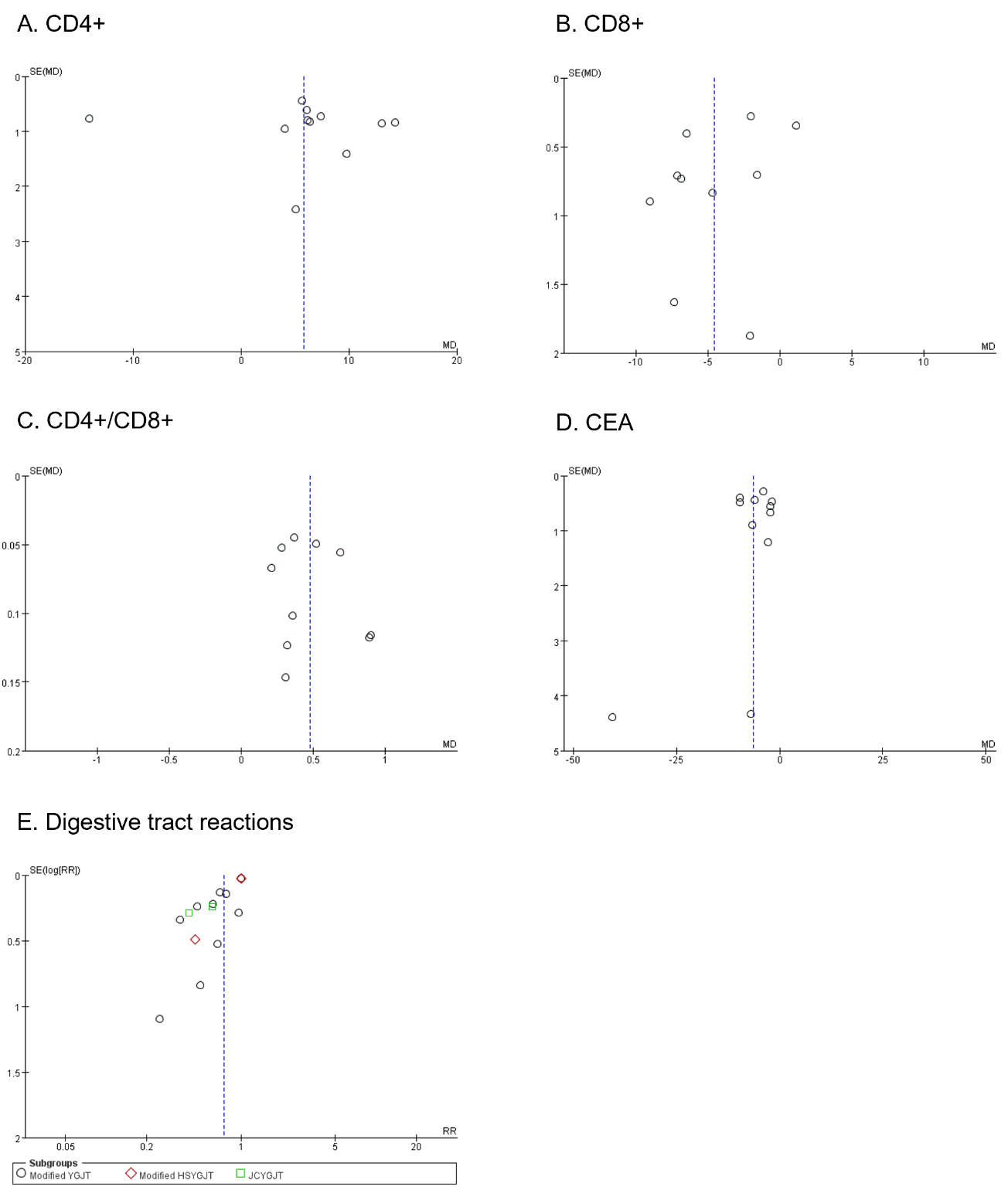

Supplement: Supplementary file 1 [file Supplementaryfile1.docx]
